# Large-scale analysis of sheep rumen metagenome profiles captured by reduced representation sequencing reveals individual profiles are influenced by the environment and genetics of the host

**DOI:** 10.1186/s12864-023-09660-3

**Published:** 2023-09-18

**Authors:** Melanie K. Hess, Hannah E. Hodgkinson, Andrew S. Hess, Larissa Zetouni, Juliana C. C. Budel, Hannah Henry, Alistair Donaldson, Timothy P. Bilton, Tracey C. van Stijn, Michelle R. Kirk, Ken G. Dodds, Rudiger Brauning, Alan F. McCulloch, Sharon M. Hickey, Patricia L. Johnson, Arjan Jonker, Nickolas Morton, Shaun Hendy, V. Hutton Oddy, Peter H. Janssen, John C. McEwan, Suzanne J. Rowe

**Affiliations:** 1 AgResearch Ltd., Invermay Agricultural Centre, Private Bag 50034, Mosgiel, 9053 New Zealand; 2 Agriculture, Veterinary & Rangeland Sciences, University of Nevada-Reno, 1664 N. Virginia St. Mail stop 202, Reno, NV 89557 USA; 3 Wageningen University & Research, P.O. Box 338, 6700 AH Wageningen, The Netherlands; 4 Graduate Program in Animal Science, Universidade Federal do Pará (UFPa), Castanhal, Brazil; 5 NSW Department of Primary Industries, University of New England, Armidale, 2351 Australia; 6 AgResearch Ltd., Grasslands Research Centre, Private Bag 11,008, Palmerston North, 4410 New Zealand; 7 AgResearch Ltd., Ruakura Research Centre, Private Bag 3115, Hamilton, 3214 New Zealand; 8 Te Pūnaha Matatini, University of Auckland, Auckland, 1010 New Zealand

**Keywords:** Genotyping-by-sequencing, Restriction enzyme reduced representation sequencing, Rumen microbiome, Metagenome, Genetics, Reference based, Reference free

## Abstract

**Background:**

Producing animal protein while reducing the animal’s impact on the environment, e.g., through improved feed efficiency and lowered methane emissions, has gained interest in recent years. Genetic selection is one possible path to reduce the environmental impact of livestock production, but these traits are difficult and expensive to measure on many animals. The rumen microbiome may serve as a proxy for these traits due to its role in feed digestion. Restriction enzyme-reduced representation sequencing (RE-RRS) is a high-throughput and cost-effective approach to rumen metagenome profiling, but the systematic (e.g., sequencing) and biological factors influencing the resulting reference based (RB) and reference free (RF) profiles need to be explored before widespread industry adoption is possible.

**Results:**

Metagenome profiles were generated by RE-RRS of 4,479 rumen samples collected from 1,708 sheep, and assigned to eight groups based on diet, age, time off feed, and country (New Zealand or Australia) at the time of sample collection. Systematic effects were found to have minimal influence on metagenome profiles. Diet was a major driver of differences between samples, followed by time off feed, then age of the sheep. The RF approach resulted in more reads being assigned per sample and afforded greater resolution when distinguishing between groups than the RB approach. Normalizing relative abundances within the sampling Cohort abolished structures related to age, diet, and time off feed, allowing a clear signal based on methane emissions to be elucidated. Genus-level abundances of rumen microbes showed low-to-moderate heritability and repeatability and were consistent between diets.

**Conclusions:**

Variation in rumen metagenomic profiles was influenced by diet, age, time off feed and genetics. Not accounting for environmental factors may limit the ability to associate the profile with traits of interest. However, these differences can be accounted for by adjusting for Cohort effects, revealing robust biological signals. The abundances of some genera were consistently heritable and repeatable across different environments, suggesting that metagenomic profiles could be used to predict an individual’s future performance, or performance of its offspring, in a range of environments. These results highlight the potential of using rumen metagenomic profiles for selection purposes in a practical, agricultural setting.

**Supplementary Information:**

The online version contains supplementary material available at 10.1186/s12864-023-09660-3.

## Background

There is increasing interest in livestock production traits relating to the impact of an individual animal on the environment, such as the amount of feed consumed, or the amount of methane emitted. This is largely due to the recognition that more sustainable methods of producing animal protein are needed to be able to meet both short- and long-term global demands.

Traits focused on the efficiency of the animal (e.g., residual feed intake) and greenhouse gas emissions (e.g., methane) are related to each other and are under host genetic control [1]. The host’s rumen microbiome plays a vital role in converting consumed feed into short chain fatty acids, which provide energy to the host but also produce methane as a by-product. Thus, there is also a link between the host microbiome and these traits [2–8]. Furthermore, recent studies have demonstrated that the microbial profile of an individual is under host genetic control [9, 10]. This indicates the potential use of the microbiome for achieving sustained changes in environmentally important traits through genetic selection, as well as its use as a management tool to identify animals likely to have a favorable or unfavorable carbon footprint. However, the microbiome composition is also controlled by the rumen environment and nutrition due to the animal’s diet [11], and separating these environmental factors from the permanent genetic signal will improve genetic selection accuracy and progress.

A high-throughput and low-cost method is needed to achieve industry-wide implementation of metagenome profiles into livestock production. Hess et al. [12] recently developed a restriction enzyme-reduced representation sequencing (RE-RRS) approach that is a cost-effective and efficient method for generating metagenome profiles on thousands of individuals. They presented two methods for generating profiles from RE-RRS sequences: a reference based (RB) approach that uses a reference database to assign reads to taxa, and a tag-based reference free (RF) approach that captured a greater proportion of the reads. However, the advantages of these two approaches to metagenome profiling need to be further explored on a larger dataset from a broader range of environmental conditions and genetic backgrounds. Furthermore, there are still many questions on how to use the data from this approach as a tool in the livestock industry. Gaining knowledge on the impact of systematic (e.g., sequencing) effects on the interpretation of the results, and the impact that life history (e.g., diet or age) plays on microbial composition will aid in the development of appropriate methods to standardize data for downstream analyses. Additionally, assessments of the interactions between host genetics and microbial composition will allow a better understanding of the role host genetics plays in influencing rumen microbial composition and how this relates to traits of interest.

To gain a better understanding of the factors that affect rumen metagenome profiling, we explore the similarities and differences in rumen metagenome profiles from samples taken in eight different conditions, with groups differing by diet, age, time off feed prior to sampling, and country. First, we assessed the practicality of using RE-RRS for high-throughput metagenome sequencing, including identification of systematic and biological impacts on metagenome profiles. We then evaluated the differences between metagenome profiles in the eight different groups through their taxonomic abundances and network diagrams. Finally, we obtained heritability and repeatability estimates for a range of genera to evaluate host genetic control of the rumen microbiome. This study highlights critical considerations when attempting to predict traits using metagenome profiles.

## Results

### Sequencing, quality control and systematic effects

RE-RRS was used to sequence 3,971 New Zealand and 508 Australian sheep rumen samples. These samples were split into eight Groups according to recorded parameters associated with each sample, i.e., age and diet of the sheep at the time of rumen sample collection, length of time the sheep was off feed before the rumen sample was taken, and country the animal lived in (Table 1 and Additional File 1). Most individuals had samples collected as part of multiple Groups, with 3.3 ± 1.4 samples per individual for New Zealand sheep and 1 ± 0 samples per individual for Australian sheep. The largest Groups were GAS and GLS, representing adults and lambs, respectively, that were on a grass (ryegrass-based pasture) diet and whose samples were collected after a short time off feed (2–4 h). Each Group was further separated into Cohorts, with individuals from the same flock and age who had their rumen samples collected over the same 1–3 day period allocated to the same Cohort. More information regarding the samples can be found in Additional File 1.

**Table 1:** Number of rumen samples and sheep by group

Group	Diet^a^	Age^b^	Time off Feed^c^	Feeding	Number of Samples	Number of Sheep
GLS	Grass	Lamb	Short	ad libitum	1074	1074
GAS	Grass	Adult	Short	ad libitum	1080	1080
GLL	Grass	Lamb	Long	ad libitum	186	93
GAL	Grass	Adult	Long	ad libitum	94	94
LLS	Pellets (L)	Lamb	Short	ad libitum	985	985
LLL	Pellets (L)	Lamb	Long	2 × MEM^d^	376	188
MAS	Pellets (M)	Adult	Short	ad libitum	176	176
AUS	Australian	Adult	Short	1.5–1.6 × MEM^d^	508	508
Total					4479	1708^e^

^a^Grass: ryegrass-based pasture. Pellets (L): lucerne pellets. Pellets (M): maintenance pellets. Australian: Chaffed lucerne and cereal hay

^b^Animal considered an Adult at ~ 15 months of age

^c^Duration between access to feed and rumen sampling: Short = 2–4 h; collected in the South Island of New Zealand or in Australia. Long = 15–16 h; collected in the North Island of New Zealand

^d^MEM: Metabolizable energy requirements for maintenance

^e^Some individuals had rumen samples collected as part of multiple Groups, although all Australian sheep only had one rumen sample

Table 2 summarizes metrics from sequencing, quality control and metagenome profiling based on the RB and RF pipelines described by Hess et al. [12], and Additional File 2 contains these metrics split by Group. The RB and RF pipelines were developed to be very high-throughput and generate metagenome profiles very efficiently. The RB pipeline involved aligning reads to a reference database consisting of the Hungate1000 Collection [13] plus four *Quinella* genomes [14], and assigning reads at the genus level. The RF pipeline involved generating a set of tags: unique 65 bp reads, starting from the cut site, that are present in at least 25% of samples [12]. Tags were generated using the full set of samples (Tag Set = “All”) for Table 2 and generated within each Group in Additional File 2. Read lengths ranged from 40 to 92 bp for all samples after quality control and removal of the adapter and barcode sequences. The proportion of reads assigned (assignment rate) for the RB approach was significantly lower than the assignment rate for the RF approach (*p*-value < 2.2 × 10^–16^, paired t-test); however, 195 of the 4479 samples (4%) had a RB assignment rate that was marginally higher than the RF assignment rate when considering tags generated on the full set of samples. Most of these samples (171/195) were from the AUS Group, which are expected to be different to the other Groups due to country, diet, and breed of the sheep. The Australian samples made up ~ 11% of the dataset, so any microbes that are unique to the Australian dataset would not be captured in the set of tags, given that tags need to be present in 25% of samples. When considering only tags generated within the AUS Group, there were only 7 that had a greater proportion of reads assigned using the RB than RF approaches.

**Table 2:** Sequencing statistics across all samples

Metric	Parameter	Mean ± SD
Post-QC sequencing^a^	Reads per sample (thousands)	713 ± 215
	Average read length (bp)	80.8 ± 1.5
	Median read length	91.6 ± 0.5
	Percent of reads ≥ 65 bp	79.3 ± 3.3%
Reference-based	Assignment rate^b^	20.8 ± 3.5%
Reference-free	Number of tags^c^ (thousands)	186
	Assignment rate^c^	29.2 ± 3.8%

^a^After demultiplexing and trimming; QC: Phred quality score of at least 20 and read length of at least 40 bp

^b^Percent of reads that were assigned to the reference database, consisting of the Hungate1000 Collection plus four *Quinella* genomes

^c^Percent of reads that were assigned to a tag, i.e., unique 65 bp reads, starting from the cut site, that are present in at least 25% of all samples

All sequencing metrics were similar across the different Groups (Additional File 2). Assignment rates for the RB pipeline ranged from 18.4% (LLS) to 23.7% (AUS). The number of tags ranged from 135k (MAS) to 264k (GAS), and the RF assignment rates ranged from 33.6% (LLS, 206k tags) to 39.6% (LLL, 228k tags). In total there were 571,479 tags identified in at least one of the Groups.

#### Systematic effects

Biological and systematic effects were evaluated for their contribution to variation in the metagenome profiles using the New Zealand samples (Table 3 and Additional File 3). Systematic effects relate to factors associated with the sample processing and sequencing approach. The effects of Library (i.e., the set of ~ 370 samples that were sequenced simultaneously) explained a significant percent of sample-to-sample variation in the number of reads, and the proportion of reads assigned using the RB and RF approaches. The Library effect was much larger for the proportion of reads assigned using the RF approach than for the other two profiling metrics (Table 3). The library effect explained 2.4 ± 5.2% of the variation in log_10_ relative abundances of each of the microbes from the RB profiles (Additional File 3). The genus *Quinella* was a major outlier in terms of the proportion of the variation that library explained (25.4%) and, with this genus removed, the library effect explained 2.0 ± 4.3% of the variation in log_10_ relative abundances.

**Table 3:** Percent variance explained by systematic and host-specific biological factors in New Zealand Sheep Rumen Samples

Profiling Metric	Group^a^	Cohort^b^	Library^c^	BatchShelf^d^	Well^e^	BRR^f^	AOD^g^	BDEV^h^
Number of Reads	3.74***	3.79***	4.08***	13.94***	16.44***	0.07	0.01	0.02
RB_Prop	15.18***	22.67***	6.66***	7.25**	10.36	0.14	0.05	0.30
RF_Prop	8.77***	28.27***	34.00***	7.66**	10.23	0.42	0.01	0.09

^a^Group = the combination of diet, age, time off feed, and country

^b^Cohort = the set of samples that were collected during the same period

^c^Library = the set of samples that were sequenced simultaneously

^d^BatchShelf = the sample freeze-drying and grinding batch, and freeze-drying shelf

^e^Well = the position on the DNA extraction plate

^f^BRR = birth rear rank, i.e., the combination of the number of lambs born to the dam at the time the lamb was born, and the number of lambs that were reared

^g^AOD = age of dam at time the individual was born

^h^BDEV = birth date deviation within contemporary group

*P*-values from an F-test

^*^ < 0.05

^**^ < 0.001

^***^ < 0.00001

Batch and shelf were related to the process of freeze drying the samples, and the combined BatchShelf effect was found to explain a significant percentage of the variation of all sequencing metrics, with the largest variation being explained in the total number of reads followed by proportion of reads assigned using the RF approach (Table 3). BatchShelf effects explained 6.1 ± 1.6% of the variation in log_10_ relative abundances of the 61 genera in the reference database, with a significant effect for 25 genera (Additional File 3). The largest BatchShelf effect was observed for *Methanobrevibacter*.

Well effects refer to the position of each sample on the plate during DNA extraction and library preparation and explained the most variation for the number of reads. Well explained approximately 10.3% of the variation in the proportion of reads for the RB and RF pipelines, although this was not significant (Table 3). Part of the reason Well effects explained a large proportion of the variation of these sequencing metrics is because there were 96 levels of this effect. Out of all the genera in the reference database, Well effects were only significant for *Quinella*, *Staphylococcus* and *Succinivibrio*, explaining 19.4%, 12.5% and 11.9% of the variation in the number of reads assigned, respectively (Additional File 3).

#### Biological effects

Group (the combination of age, feed, time off feed and country) and Cohort (the set of animals from the same flock that were sampled during the same 1–3 day period) effects explained a significant proportion of the variation in the number of reads, proportion of reads assigned using the RB and RF approaches (Table 3), as well as the log_10_ relative abundances of all taxa in the reference database (Additional File 3). Of the three profiling metrics, Group effects explained the most variation in the proportion of reads assigned using the RB approach. Cohort effects explained over 20% of the variation for each of the profiling metrics. Group effects explained the most variation in *Selenomonas* (60.6%), *Anaerovibrio* (43.8%) and *Sarcina* (41.4%), while Cohort effects explained the most variation in *Ruminobacter* (49.1%), *Cellulomonas* (37.9%) and *Succinivibrio* (33.2%).

Biological effects of birth rear rank (BRR), age of dam (AOD) and birth date deviation (BDEV) did not have significant effects on the number of reads or the proportion of assigned reads using either the RB or RF approaches (Table 3). BRR, AOD or BDEV explained a small amount of the variation in log_10_ relative abundance for some of the taxa, but the largest effect was that of BRR on the log_10_ relative abundance of *Sarcina*, and this explained only 1.15% of the variation (Additional File 3).

### Reference-free tags

#### Comparison by Group

**Fig. 1:**
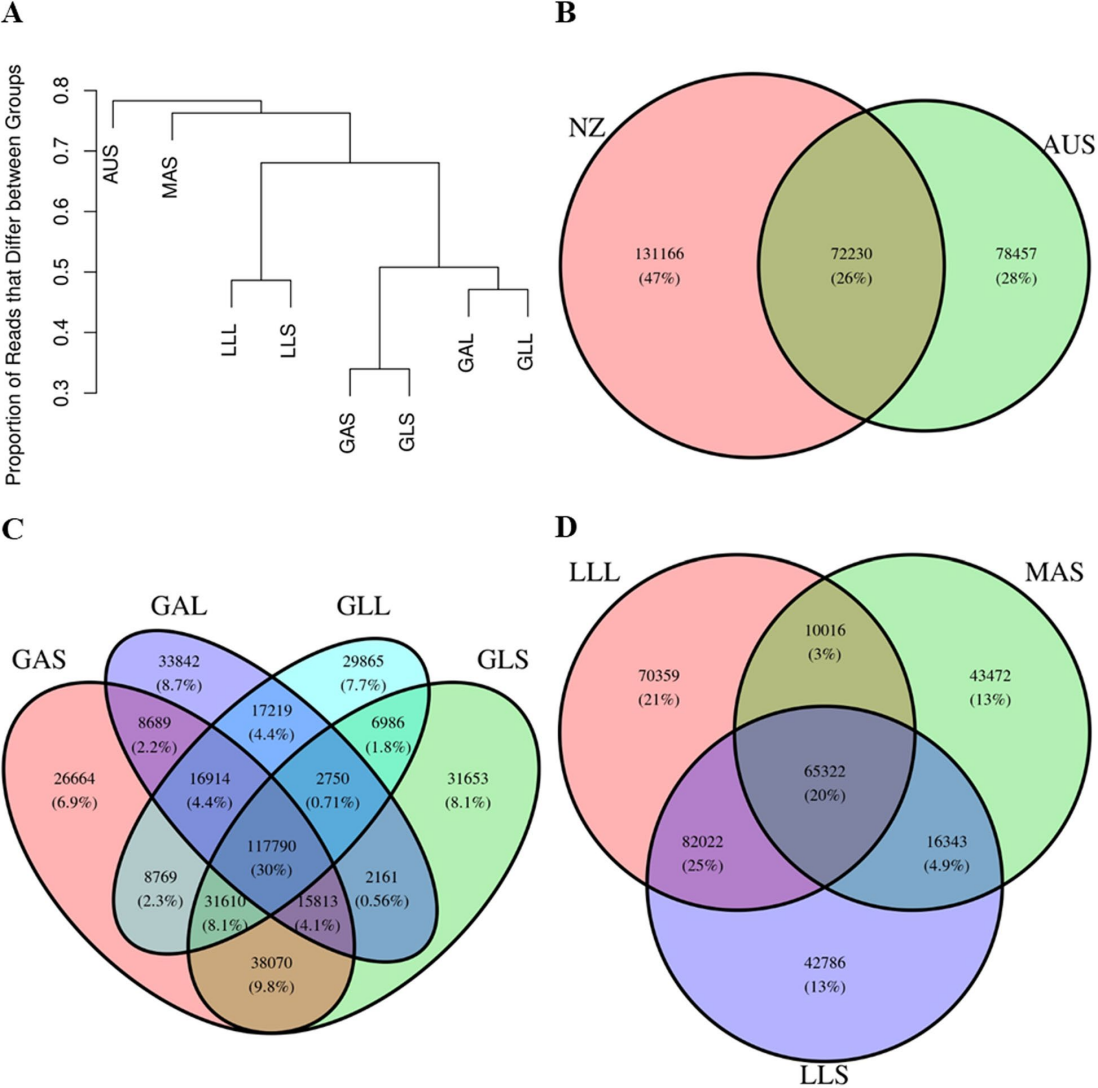
Comparison of reference-free tags by Group. All tags were generated based on tag prevalence of 25% within each Group. **A**: Dendrogram of the proportion of tags that differ between Groups. **B**: Comparison of tags between New Zealand (NZ) and Australian (AUS) sheep rumen samples. **C**: Comparison of tags between samples collected when the sheep was grazing pasture (GAS: adults grazing pasture and sampled a short -time off feed, GAL: adults grazing pasture and sampled a long -time off feed, GLL: lambs grazing pasture and sampled a long time off feed, and GLS: lambs grazing pasture and sampled a short time off feed). **D**: Comparison of tags between samples collected when the sheep was fed pellets (LLL: lambs fed lucerne pellets and sampled a long time off feed, LLS: lambs fed lucerne pellets and sampled a short time off feed, and MAS: adults fed maintenance pellets and sampled a short time off feed)

The main set of tags that was used for the analyses in this paper is the set of “All” tags – those generated using all 4,479 samples. This tag set enables comparison between all samples for the given analyses. We were also interested in how similar the tags from different Groups would be. Therefore, we generated tag sets within each of the groups (i.e., tags present in 25% of samples within each of the Groups), as well as a tag set for the New Zealand samples. Fig. 1 compares the similarity of the tag sets generated from the samples in each the different Groups (i.e., Group-specific tag sets). A hierarchical clustering analysis demonstrated that the Australian samples cluster separately from the New Zealand (NZ) samples, which is to be expected due to the different environments and diets between the two countries (Fig. 1A). The NZ samples were further separated by diet, followed by the time off feed. The lowest clades, representing the most similar tag sets, separated Groups by the age of the sheep sampled (i.e., lamb or adult), within diet and time off feed. A comparison of the tag sets between NZ and Australia (Fig. 1B) showed that most tags were different between NZ and Australian samples, however there were still over 70,000 tags in common between the two countries. Over 117,000 tags were shared between tag sets from sheep that were grazing pasture (Fig. 1C). Consistent with the hierarchical analysis in Fig. 1A, more tags were found in common between GAS and GLS Groups than any other pair (Fig. 1C). A comparison of tag sets between Groups that were fed a pellet diet (Fig. 1D) showed that tags from sheep fed the same type of pellets (lucerne pellets) at the same age (lambs) were more similar than the Group of adults fed a different type of pellet (maintenance). Overall, these results show that diet, age, and.

#### Taxonomies of reference-free tags

Tags were assigned to taxa based on comparison against the *Ovis aries* 3.1 genome assembly (OAR 3.1), the RB reference database (consisting of genomes from the Hungate1000 Collection [13] plus four *Quinella* genomes [14]) and GenBank [15] to discover what additional organisms were being captured by RF metagenome profiles beyond the RB microbial profiles. Table 4 shows the proportion of tags that were able to be assigned at the various taxonomic levels for the different sets of tags explored in this study. Most tag sets had approximately 40% of tags assigned at any taxonomic level, and the set of tags that were present in All Groups (i.e., tags present in all eight Groups) had the highest assignment rate, with taxonomy assigned for 47.7% of tags. Between 30 and 35% of tags were typically assigned at the genus level, with the greatest rate for those in All Groups at 41.8%.

**Table 4:** Percent of tags that are assigned at different taxonomic levels for the different tag sets

Tag Set^a^	Number of Tags	Kingdom	Phylum	Class	Order	Family	Genus	Species
GLS	246,833	40.9%	37.7%	37.0%	36.7%	35.1%	34.1%	22.9%
GAS	264,319	41.4%	38.2%	37.5%	37.3%	35.8%	34.8%	22.5%
GLL	231,903	42.5%	39.3%	38.6%	38.4%	37.0%	36.1%	22.9%
GAL	215,178	43.3%	40.4%	39.7%	39.5%	38.0%	37.2%	22.7%
LLS	206,473	38.6%	35.7%	34.9%	34.7%	33.1%	32.3%	20.7%
LLL	227,719	37.7%	35.1%	34.4%	34.1%	32.6%	31.8%	20.9%
MAS	135,153	40.2%	37.0%	36.3%	36.0%	34.6%	33.8%	21.7%
AUS	150,687	41.5%	38.1%	37.4%	37.2%	35.9%	35.1%	20.7%
All	186,302	42.0%	38.7%	38.0%	37.8%	36.3%	35.5%	22.4%
NZ	203,396	41.4%	38.3%	37.5%	37.3%	35.8%	35.0%	22.4%
All Groups	21,119	47.7%	44.7%	44.0%	43.9%	42.4%	41.8%	21.7%
All Tags	571,479	38.8%	36.0%	35.2%	34.9%	33.5%	32.6%	21.7%

All Tags is the set of tags that are present in the tag sets for at least one of the eight Groups

^a^Tags present in 25% of samples in the GLS, GAS, GLL, GAL, LLS, LLL, MAS and AUS Groups; all samples (All) or New Zealand samples (NZ). All Groups is the set of tags that are present in the tag sets of all the eight Groups

**Fig. 2:**
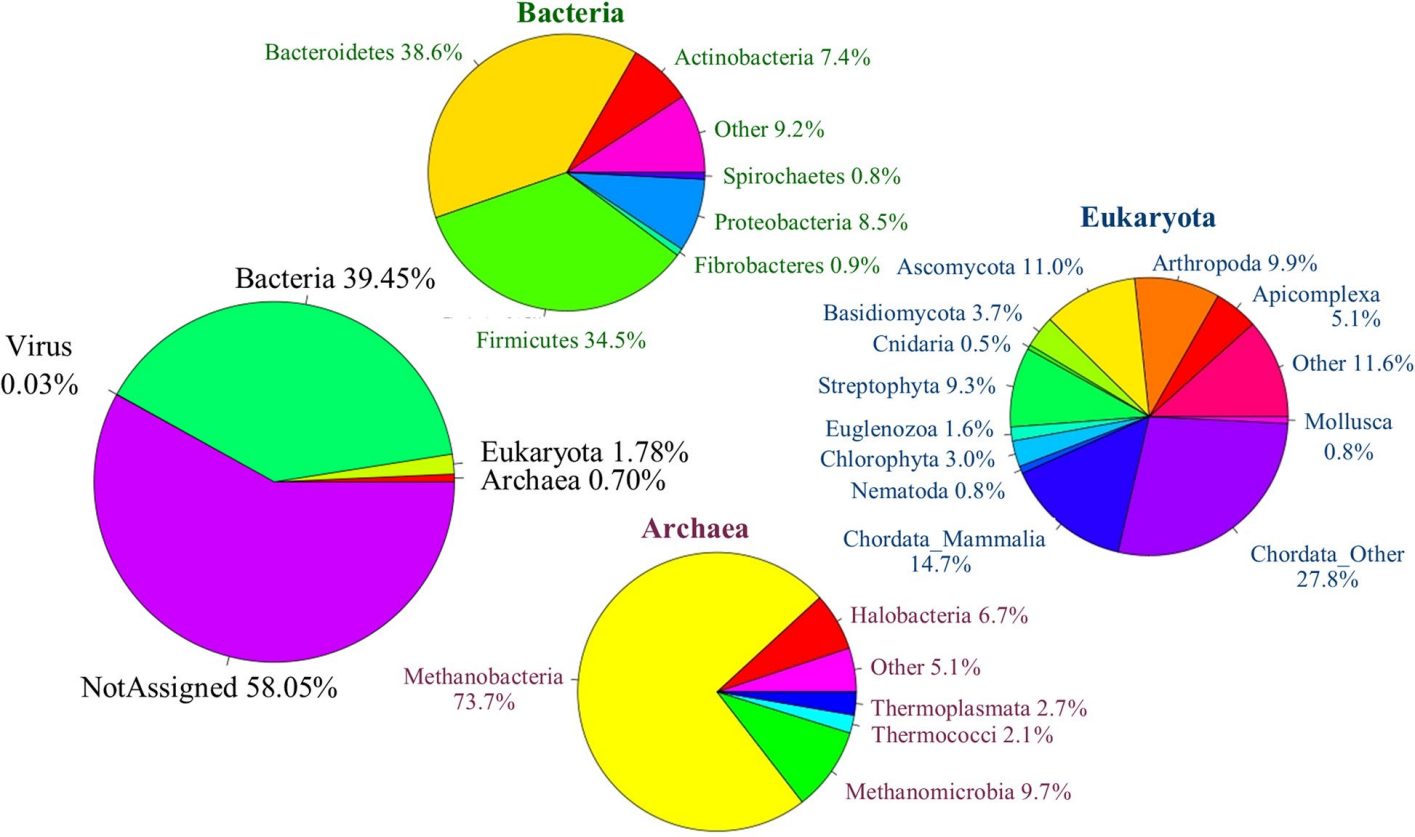
Taxonomies of reference-free tags. Tags were assigned taxonomy based on comparison to GenBank, the Hungate1000 Collection and the sheep genome (Eukaryota ➔ Chordata_Mammalia). This figure shows the taxonomy of tags at the domain level, within Bacteria and Eukaryota at the phylum level, and within Archaea at the class level. Taxa were included in the Bacteria, Archaea and Eukaryota charts if they were identified for at least 0.5% of tags assigned to that kingdom. Graphs show the proportion of tags assigned to each taxonomic level and do not reflect the relative abundance of each tag

Fig. 2 shows the taxonomic assignment of tags based on comparison against the GenBank and Hungate1000 Collection genomes, as well as alignment against the sheep genome (OAR 3.1). Less half the tags were able to be assigned at any taxonomic level and most of those were assigned to the domain Bacteria, followed by Eukaryota and then Archaea. Within Bacteria, most tags were assigned to the phyla *Bacteroidetes* and *Firmicutes*. The majority of Eukaryota tags were from the phylum *Chordata*, of which 34.5% were assigned to a mammalian genome. Archaeal tags were dominated by methanogens, with 75% of tags assigned to *Methanobacteria*, followed by *Methanomicrobia* at just under 10%.

time off feed when sampled are major drivers of the rumen metagenome.

### Relative abundances of bacteria and archaea

**Fig. 3:**
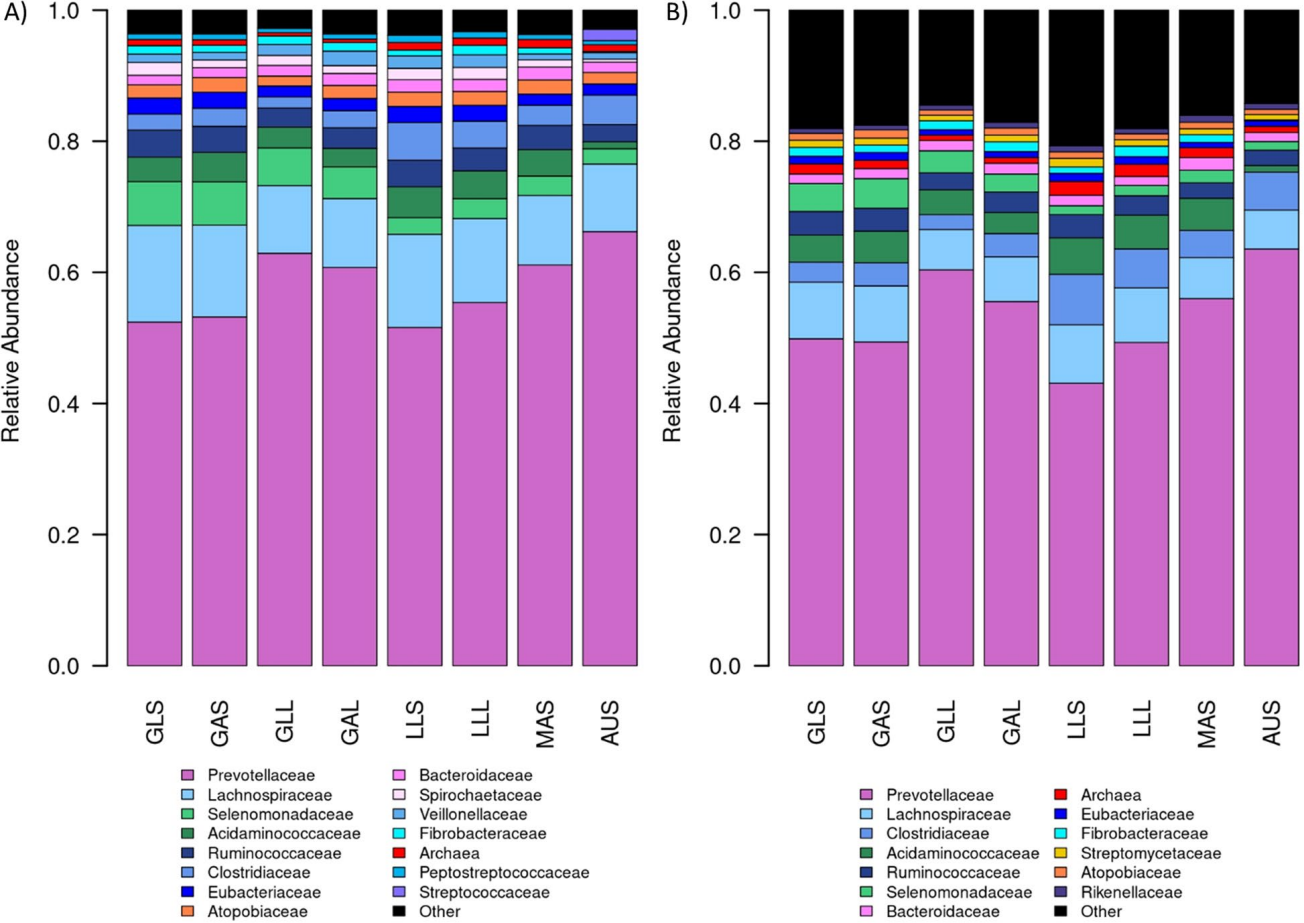
Relative abundances of bacterial and archaeal taxa from reference-based (A) and reference-free (B) metagenome profiles. **A**: Family level taxa relative abundances for reference-based (RB) microbial profiles obtained by alignment with the Hungate1000 Collection. **B**: Family level taxa relative abundances for reference-free (RF) microbial profiles obtained by alignment of RF tags to GenBank and the Hungate1000 Collection, considering only bacterial and archaeal taxonomies from the set of tags generated on all samples. Taxa with an average abundance less than 1% in all of the Groups were combined into “Other”

Microbial profiles were generated for the RF metagenome profiles by considering only bacterial and archaeal taxa from the tags that were assigned at the genus level in the previous section. Fig. 3 shows the relative abundances of the RB and RF microbial profiles at the family level, with those with an abundance of < 1% in all Groups combined into the “Other” category for visualization purposes. Overall, the microbial profiles were similar, however, there were some differences. There were four families that were present in the RB plot (Fig. 3A) that were absent in the RF plot (Fig. 3B): *Peptostreptococcaceae*, *Spirochaetaceae*, *Streptococcaceae* and *Veillonellaceae*. *Peptostreptococcaceae*, *Spirochaetaceae* and *Veillonellaceae* had low abundance and only just met the 1% threshold for the RB plot, but were below the 1% threshold for the RF approach and are captured in the “Other” category of Fig. 3B. *Streptococcaceae* was most abundant in the AUS Group of samples for the RB pipeline and therefore it is possible that it was omitted in the RF pipeline due to *Streptococcaceae* tags not reaching the 25% prevalence threshold when considering the full set of samples. *Rikenellaceae* and *Streptomycetaceae* were present in the RF plot (Fig. 3B) but not the RB plot (Fig. 3A). This was because these families are not captured within the reference database used for the RB analysis. The “Other” category made up a much larger portion of the microbial profile for each Group in the RF plot (Fig. 3B) than the RB plot (Fig. 3A), as there were a large number of additional families that are present at low levels (< 1% abundance) in the RF microbial profiles.

*Prevotellaceae* was the most abundant family in all Groups, representing 52% to 66% of RB microbial profiles and 43% to 64% of the RF bacterial or archaeal microbial profiles (Fig. 3). *Lachnospiraceae* was the second most abundant family, representing 10% to 15% of RB microbial profiles and 6% to 9% of RF bacterial or archaeal profiles. Consistent with the comparison of the tag sets between Groups (Fig. 1), profiles for lambs and adults on the same diet were very similar to each other (Fig. 3), with the largest differences being driven by time off feed and diet. This indicates that the differences in profiles between Groups encompass both the prevalence and abundance of taxa. Pellet diets (LLS, LLL and MAS) tended to have the highest proportions of archaea, with the profiles from grass fed sheep taken a long time off feed (GAL and GLL) having the lowest abundance of archaea.

### Relationships between samples from different Groups

#### Reference-based relationships

**Fig. 4:**
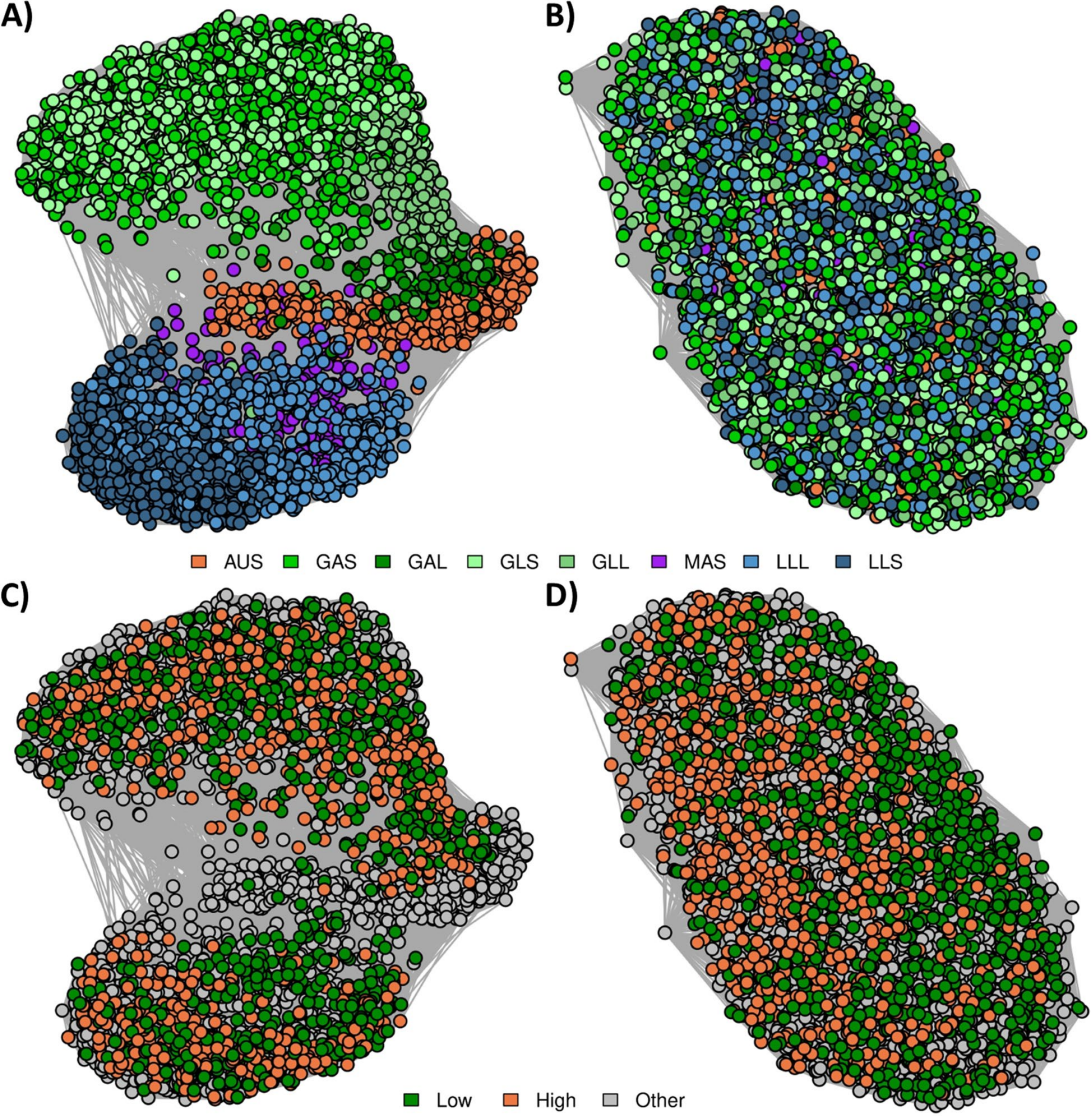
Reference-based (RB) relationships between samples by Group or selection line. **A**: log_10_ normalized RB profiles coloured by Group. **B**: Cohort normalized RB profiles coloured by Group. For A and B, the colours were based on diet: sheep fed a grass diet (green), sheep fed a lucerne pellet diet (blue), sheep fed a maintenance pellet diet (purple), and Australian sheep fed a chaffed lucerne and cereal hay diet (orange). **C**: log_10_ normalized RB profiles coloured by methane selection line. **D**: Cohort-normalized RB profiles coloured by methane selection line. For C and D, colours were based on whether the sheep were from the low methane line (green), the high methane line (orange), and from the other flocks (grey). Edges represent the correlation between the samples from the log_10_ normalized or cohort-adjusted microbial relationship matrix

Network diagrams were generated using log_10_ normalized (Fig. 4A) and Cohort-adjusted (Fig. 4B) RB microbial profiles to visualize the impact of diet, age, and time off feed on these two normalization methods. A set of individuals were from sheep selection lines, selected for high or low methane emissions [16–18], which allowed us to evaluate whether samples clustered based on selection line (Fig. 4C and D). A clear effect of Group was present for log_10_ normalized RB microbial profiles, and this effect was largely driven by the different diets the individuals were fed when the samples were collected (Fig. 4A), with no clear signal present based on methane selection line (Fig. 4C). This was unexpected, given that sheep divergent for methane emissions have different rumen microbial profiles [2]. However, normalization of each taxon within Cohort successfully removed the effects of diet, age, and time off feed (Fig. 4B), resulting in clearer separation of samples based on methane selection line (Fig. 4D).

#### Reference-free relationships

**Fig. 5:**
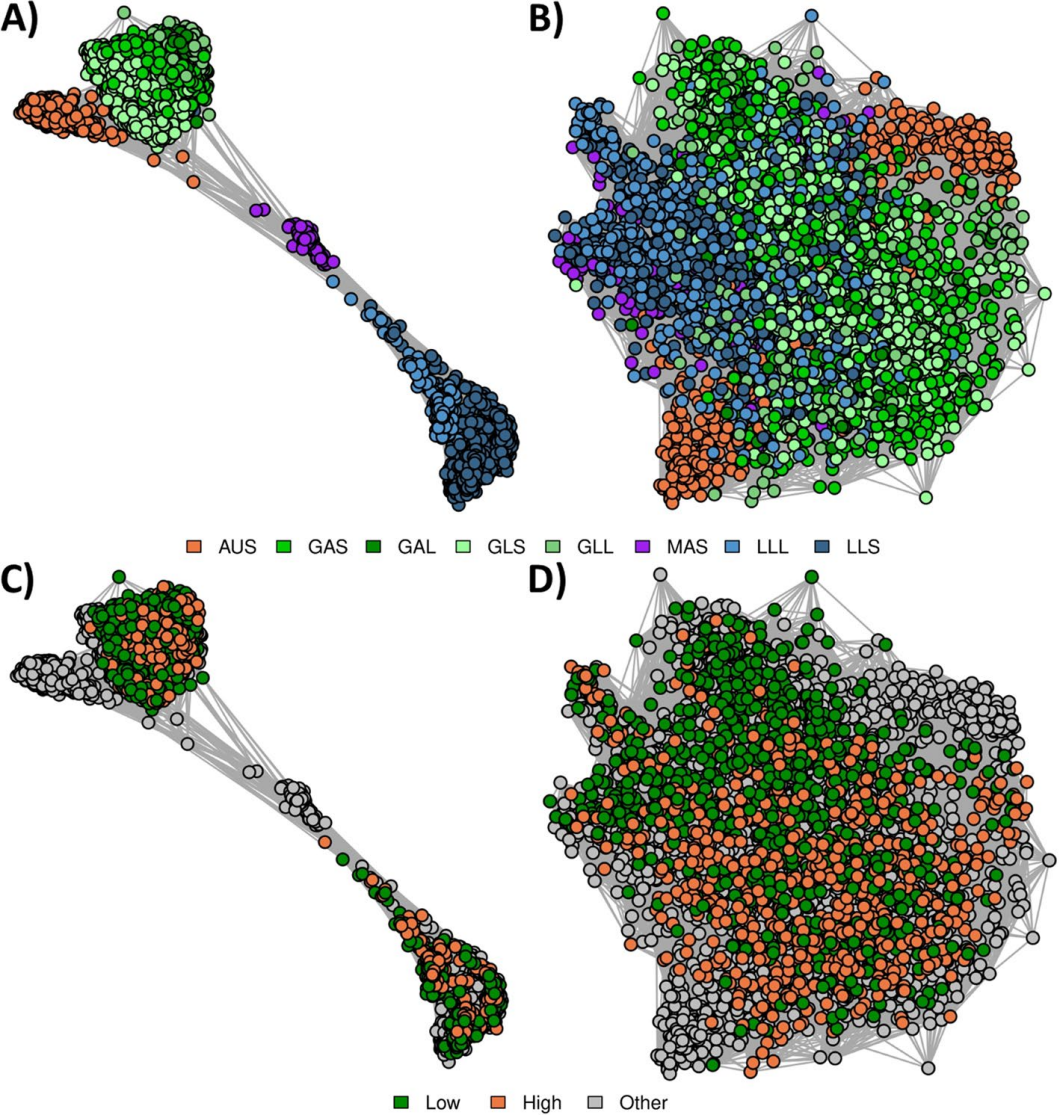
Reference-free (RF) relationships using tags generated on the full set of samples. **A**: log_10_ normalized RF profiles coloured by Group. **B**: Cohort normalized RF profiles coloured by Group. For A and B, the colours were based on diet: sheep fed a grass diet (green), sheep fed a lucerne pellet diet (blue), sheep fed a maintenance pellet diet (purple), and Australian sheep fed a chaffed lucerne and cereal hay diet (orange). **C**: log_10_ normalized RF profiles coloured by methane selection line. **D**: Cohort normalized RF profiles coloured by methane selection line. For C and D, colours were based on whether the sheep were from the low methane line (green), the high methane line (orange), or from the other flocks (grey). Edges represent the correlation between the samples from the log_10_ normalized or cohort-adjusted metagenome relationship matrix

Network diagrams were also generated from RF metagenome profiles. Fig. 5 shows network diagrams from tags generated on all samples (Tag Set “All” from Table 4), which accounted for 29.2 ± 3.8% of reads, ranging from 25.8% (AUS) to 31.6% (GLS; Additional File 2). The log_10_ normalized network diagrams (Fig. 5A and D) showed a similar pattern to those generated from the RB microbial profiles, but the RF diagrams showed that samples from the same diet were more tightly clustered and samples from different diets were more distinct from each other. Cohort adjustment of the RF profiles (Fig. 5B and D) resulted in reduced signal due to the Group effect, although some clustering in the samples due to diet remained even after the Cohort adjustment was performed. Despite the Group effect still being present, samples tended to cluster by methane selection line after Cohort adjustment (Fig. 5D).

**Fig. 6:**
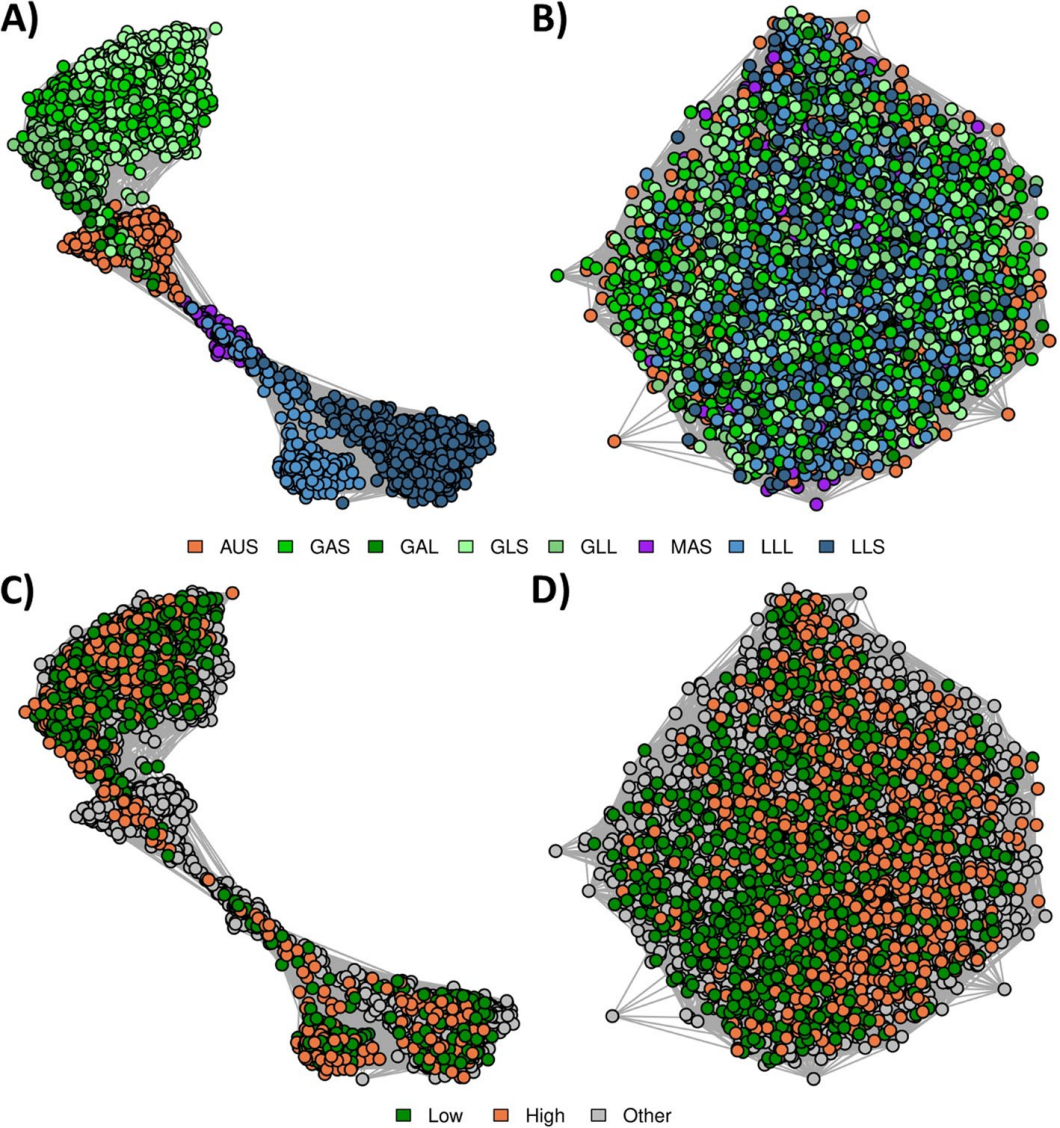
Reference-free (RF) relationships using tags assigned at genus level. **A**: log_10_ normalized RF profiles coloured by Group. **B**: Cohort normalized RF profiles coloured by Group. For A and B, the colours were based on diet: sheep fed a grass diet (green), sheep fed a lucerne pellet diet (blue), sheep fed a maintenance pellet diet (purple), and Australian sheep fed a chaffed lucerne and cereal hay diet (orange). **C**: log_10_ normalized RF profiles coloured by methane selection line. **D**: Cohort normalized RF profiles coloured by methane selection line. For C and D, colours were based on whether the sheep were from the low methane line (green), the high methane line (orange), or from the other flocks (grey). Edges represent the correlation between the samples from the log_10_ normalized or cohort-adjusted metagenome relationship matrix

Network diagrams were produced from metagenome profiles using the genus-level assignments of the set of “All” RF tags (Fig. 6) to investigate if this would reduce the clustering by Group in the Cohort-adjusted microbial profiles and allow the methane selection line signal to come through. These profiles represented 12.1 ± 2.3% of reads, ranging from 10.5% (LLS) to 13.5% (GLL; Additional File 2). The log_10_ normalized profiles clustered based on Group (Fig. 6A), consistent with RB and RF methods using all tags (Figs. 4A and 5A). However, the separation between samples on different diets was not as extreme as for the network diagrams generated using “All” tags. Clustering tags based on taxonomic assignment at the genus level successfully removed any signal due to diet, age or time of feed that was present in the set of “All” tags (Fig. 5B), and methane selection line samples clustered primarily by line (Fig. 6D), as was found in the RB network diagrams (Fig. 4).

**Fig. 7:**
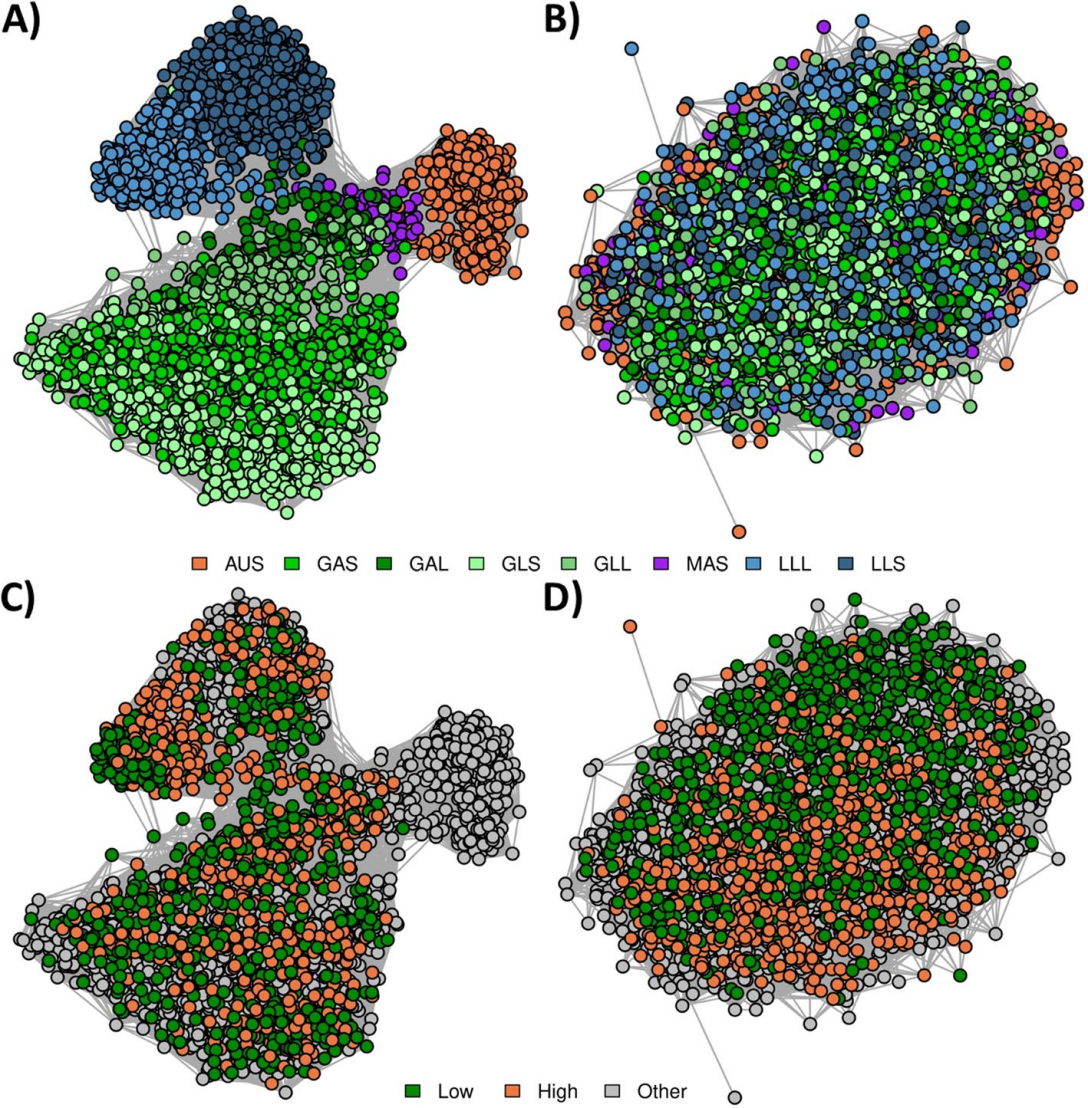
Reference-free (RF) relationships using tags assigned in all Groups. **A**: log_10_ normalized RF profiles coloured by Group. **B**: Cohort normalized RF profiles coloured by Group. For A and B, the colours were based on diet: sheep fed a grass diet (green), sheep fed a lucerne pellet diet (blue), sheep fed a maintenance pellet diet (purple), and Australian sheep fed a chaffed lucerne and cereal hay diet (orange). **C**: log_10_ normalized RF profiles coloured by methane selection line. **D**: Cohort normalized RF profiles coloured by methane selection line. For C and D, colours were based on whether the sheep were from the low methane line (green), the high methane line (orange), or from the other flocks (grey). Edges represent the correlation between the samples from the log_10_ normalized or cohort-adjusted metagenome relationship matrix

Network diagrams were produced from RF metagenome profiles generated using the “All Groups” tag set (i.e., tags were identified in all of the eight Groups) (Fig. 7) to evaluate whether this was another method that could reduce the clustering by diet that was observed in the Cohort-adjusted RF profiles (Fig. 5B). These metagenome profiles represented 8.1 ± 1.8% of reads, ranging from 7.1% (GAS) to 10.6% (MAS) (Additional File 2). These metagenome profiles (Fig. 7) resulted in very similar patterns to those for the RB profiles (Fig. 4) and the RF profiles from tags assigned taxonomy (Fig. 6) with log_10_ normalized RF profiles clustering by Group and Cohort, and adjusted RF profiles primarily clustering by methane selection line.

### PERMANOVA Analysis

A PERMANOVA analysis was run to test the significance of Group and Selection Line effects on RB and RF relative abundance metagenome profiles. The p-values from all analyses were 0.001 (Table 5), which means that the observed data was the most extreme classification of all permutations, and the RB and RF metagenome profiles differ significantly by both Group and methane selection line.

**Table 5:** Pseudo-F-Statistic and *P*-value from PERMANOVA analysis of group and selection line effects on metagenome profiles

Dataset	Parameter	Reference-Based		Reference-Free	
		Pseudo-F	*P*-value	Pseudo-F	*P*-value
All	Group	293.02	0.001	165.30	0.001
Methane Lines	Group	125.26	0.001	65.06	0.001
	Methane Line	20.74	0.001	10.53	0.001

### Heritability and repeatability estimates

#### Reference-based estimates

**Fig. 8:**
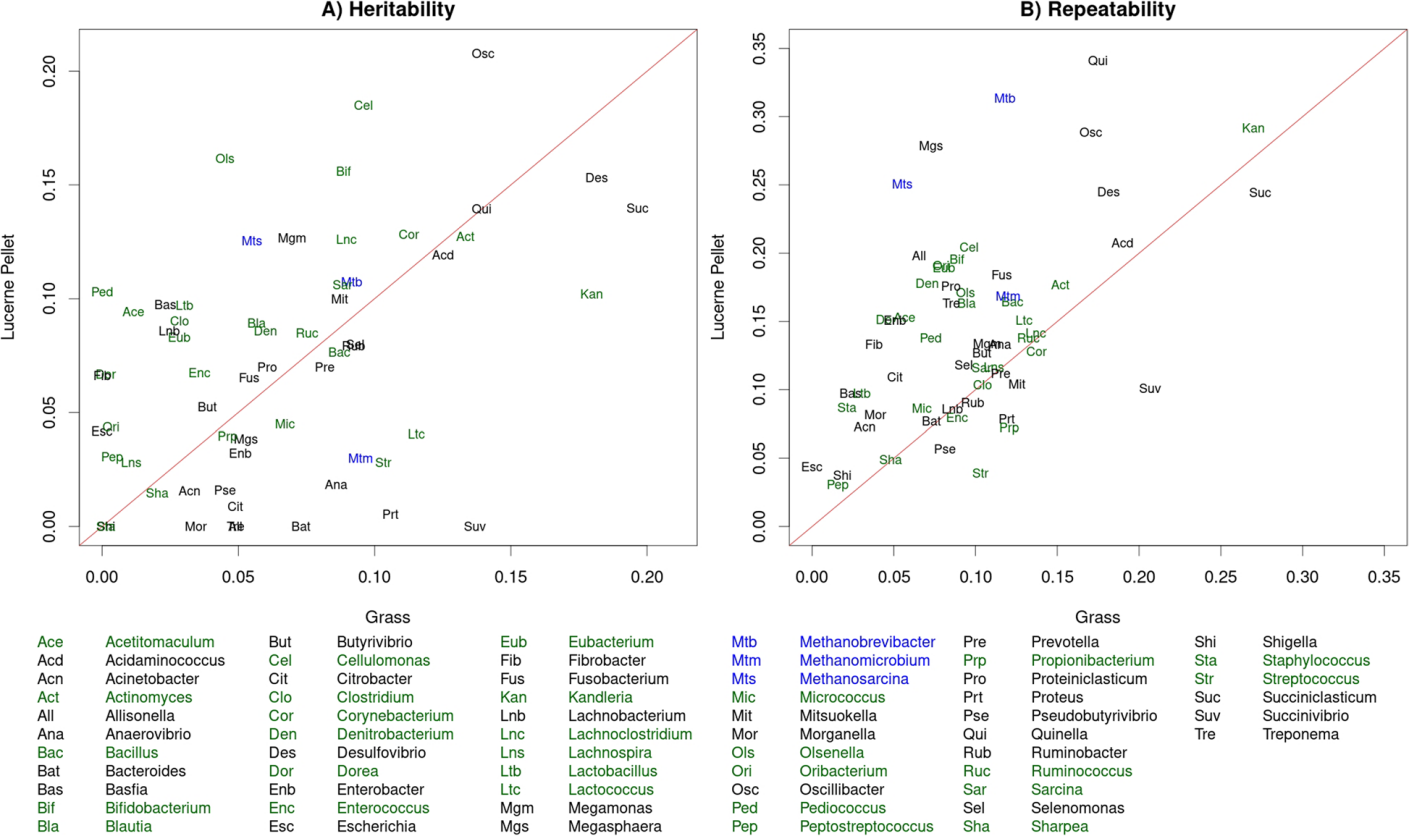
Microbial heritability (**A**) and repeatability (**B**) estimates from sheep fed a Grass vs Lucerne Pellet diet. Estimates were based on Cohort-adjusted microbial profiles using the reference-based approach. Microbes are coloured based on whether the microbe is gram-negative (black), gram-positive (green) or archaea (blue)

Heritabilities and repeatabilities for each of the genera in the reference database were estimated based on the set of samples from sheep fed grass (GAS, GLS, GLL, GAL), the set of samples from sheep fed lucerne pellets (LLL and LLS) and all New Zealand samples (Fig. 8 and Additional File 4: Supplemental Table 7). The largest heritability estimates for each set of samples were: *Succiniclasticum* (Grass, 0.20 ± 0.04), *Oscillibacter* (Lucerne Pellet, 0.21 ± 0.05) and *Desulfovibrio* (NZ, 0.14 ± 0.02), and the largest repeatability estimates for each set of samples were: *Succiniclasticum* (Grass, 0.27 ± 0.03; NZ, 0.17 ± 0.02) and *Quinella* (Lucerne Pellet, 0.34 ± 0.05). There were 10 genera that were within the top 6 most heritable genera for at least one set of samples: *Bifidobacterium*, *Cellulomonas*, *Corynebacterium*, *Desulfovibrio*, *Kandleria*, *Olsenella*, *Oscillibacter*, *Quinella*, *Succiniclasticum* and *Succinivibrio*.

The correlation of heritability estimates between each of set of samples (Grass and Lucerne Pellet, Grass and NZ, Lucerne Pellet and NZ) was higher than the correlation between repeatability estimates for the same set of samples (Fig. 8 and Additional File 4: Supplemental Table 7). The correlation in estimates between Grass and Lucerne Pellet sets was 0.44 for heritability and 0.42 for repeatability, while the correlation in estimates for the Grass and NZ sets was much higher at 0.85 for heritability and 0.79 for repeatability. The correlation in estimates between Lucerne Pellet and NZ sets was slightly lower than those between the Grass and NZ sets, at 0.71 for heritability and 0.65 repeatability.

#### Reference-free estimates

**Fig. 9:**
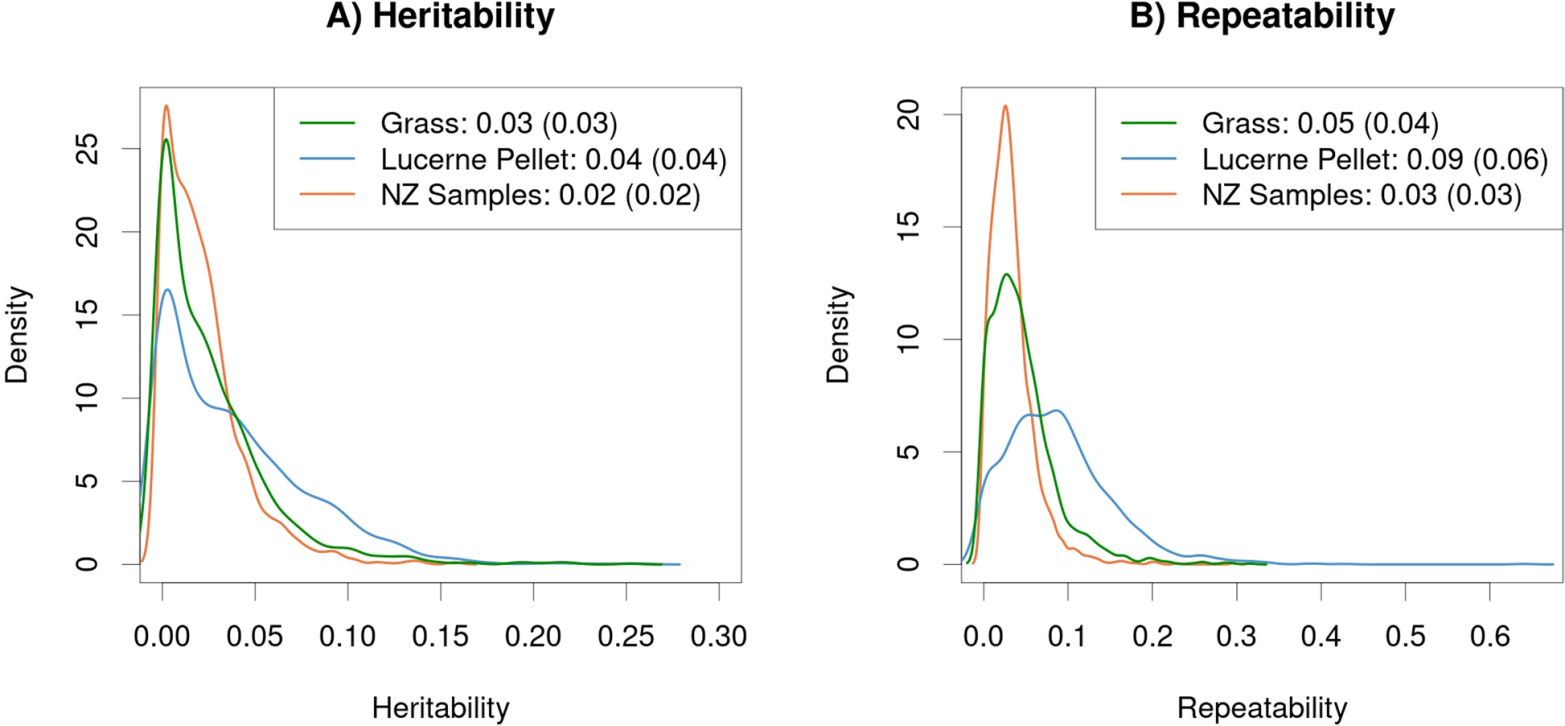
Distribution of heritability (**A**) and repeatability (**B**) estimates of reference-free tags clustered by genus. Estimates were calculated on the set of samples from sheep fed a grass diet (green), sheep fed a lucerne pellet diet (blue), and all New Zealand samples (orange). Heritability estimates were based on Cohort-adjusted metagenome profiles from the set of tags generated on all samples and assigned taxonomy at the genus level after aligning to the sheep genome, the Hungate1000 Collection and GenBank. The numbers given in the legends are the mean and standard deviation of the estimates across the 1751 genera

Heritabilities and repeatabilities were also computed for the RF approach using the “All” tag set with taxonomy assignment at the genus level. This was done for the same sets of samples as the RB heritability and repeatability analyses. Heritability and repeatability estimates tended to be highest for the Lucerne Pellet set and lowest for the NZ set (Fig. 9, Additional File 4: Supplemental Table 8). There were 13 genera that were within the top 6 most heritable genera for at least one set of samples: *Apis* (Eukaryota), *Buceros* (Eukaryota), *Dictyostelium* (Eukaryota), *Epidinium* (Eukaryota), *Ichthyophthirius* (Eukaryota), *Maricaulis* (Bacteria), *Panicum* (Eukaryota), *Pelodictyon* (Bacteria), *Perkinsus* (Eukaryota), *Quinella* (Bacteria), *Rhizophagus* (Eukaryota), *Strongyloides* (Eukaryota) and *Succiniclasticum* (Bacteria). *Quinella* and *Succiniclasticum* were the only two of these genera that were present in the reference database used for the RB approach.

As with the RB profiles, the correlation of heritability estimates between the sets of samples was higher than the repeatability estimates from the same set of samples. The correlation of estimates between the Grass or Lucerne Pellet sets was 0.34 for heritability and 0.33 for repeatability. The correlation of estimates between the Grass and NZ sets was the same as the RB profiles for heritability at 0.85 and slightly lower than the RB profiles for repeatability at 0.72. The correlation in heritability and repeatability estimates between Lucerne Pellet and NZ sets was again slightly lower than the estimates between Grass and NZ sets, at 0.63 and 0.48, respectively.

## Discussion

### High-throughput metagenome sequencing

New tools are needed to achieve the goal of producing sustainable livestock with improved feed efficiency and a reduced carbon footprint, including the integration of novel phenotypes, genomic methods, and other management tools. It is foreseeable that metagenomics is one such tool that will become invaluable to the livestock industry. Rumen microbes play a direct role in the degradation and digestion of ruminants’ feed and therefore have an impact on environmentally and economically important traits, including feed efficiency and greenhouse gas emissions, which are traits that are driven by the degradation of feed by these microbes. Furthermore, metagenome profiles can be used as a tool for genetic improvement, as well as a management tool, given that metagenome profiles are influenced by both host genetics and environmental factors. To achieve the goal of using metagenomics as an analytical tool for livestock production, methodologies need to be developed that are high-throughput and low-cost to enable industry-wide uptake.

In a recent study, Hess et al. [12] developed a method that uses the same RE-RRS scheme used in genotyping-by-sequencing to obtain metagenome profiles on individuals. They showed that this method is at least as sensitive as 16S rRNA gene sequencing, a method for which comparable datasets are available. Due to the ability to sequence at low depth without compromising the ability to capture metagenomic information, they showed that RE-RRS was suitably high-throughput for potential adoption by the livestock industry. In our current study, we applied this technique to thousands of sheep rumen samples, which allowed further assessment of this technique and afforded the opportunity to look at the major factors influencing rumen metagenome profiles in sheep. We have shown that RE-RRS is a robust method that works across rumen samples collected from sheep on a variety of diets, a range of ages, different times off feed, and diverse environments.

### Factors influencing rumen metagenome profiles

A core rumen bacterial microbiome has been observed across ruminant species, but the relative abundances of each of these microbes varies across species and diets [11]. We have shown through a variety of analyses that the environmental parameters Group (incorporating diet, age, and time off feed prior to sampling) and Cohort (the set of animals sampled at the same time) play a major role in the rumen metagenome profiles that are generated through RE-RRS, which is consistent with other studies using other microbial profiling approaches [19]. In addition to biological factors, systematic factors related to the sequencing process may impact metagenome profiles. We explored a variety of biological and systematic effects that may influence rumen metagenome profiles.

Investigation into the number of reads per sample showed variation was largely driven by the systematic effects of Library, the combined effect of freeze dryer and grinding Batch and freeze dryer Shelf, and Well position. Library effects can encompass a variety of things, such as library preparation and flow cell differences. Batch and Shelf effects encompass a variety of other effects in this analysis, as they were intentionally confounded with other “nuisance” factors, such as DNA extraction date, the person doing the DNA extraction and grinding of the rumen samples, as well as potential differences due to the shelf and batch of freeze drying. Well effects were the effect of a particular well position across all the plates in our study, and would encompass systematic effects in either the machines that do the pipetting (e.g., if one pipettes slightly more of sample A1 into the library), or due to differences in the ability of the barcodes to efficiently ligate to the sequences. In contrast, the proportion of reads assigned using the RB or RF approach was more strongly driven by biological factors such as Group and Cohort effects. An exception to this was the large impact Library had on the proportion of reads assigned using the RF approach (34%; Table 3). O’Leary et al. [20] explain that library effects can be attributed to several factors, including differences in reagents, laboratory ambient temperature, DNA quality and DNA concentration. These factors cannot be fully avoided, but randomization of samples from different treatments across libraries, as done in our study, can mitigate these effects. O’Leary et al.’s [20] description of Library effects was in reference to genotyping-by-sequencing but also applies to RE-RRS for metagenome profiling. The RF approach for metagenome profiling introduces an additional source of variation between libraries: some sequences may be common in some libraries and rarer in others, which may impact the tags that are identified, and therefore the proportion of reads captured in the RF profile. Tag selection will be influenced by the variation in samples included in a dataset, and unbalanced representation of host genotypes, diets or environments can be expected to skew tag selection. There was a moderately high correlation between the RB and RF proportion of reads assigned for a Library (Pearson = 0.663; Spearman = 0.739), however Library had a much smaller impact on the proportion of reads assigned using the RB approach compared to the RF approach (6.66% vs 34.00%; Table 3), suggesting that the large Library effect for the RF assignment rate may be due to the increased resolution of using this approach compared to the RB approach.

A distinct set of tags was generated for each Group, with some Groups showing more similar tag sets than others (Fig. 1). Groups that differed by only the age of the sheep (e.g., GAS vs GLS) had the most similar sets of tags, followed by those that were collected a short or long time off feed (e.g., GAS vs GAL), with the biggest differences in tag sets being between Groups from different diets (e.g., GAS vs MAS). The tag set generated on the Australian dataset (AUS) had the most distinct tag set, which is not surprising given the diverse environmental conditions in Australia compared to New Zealand.

In this study, time off feed was also confounded with location for the New Zealand samples, because those that were collected after only a short time off feed were from the lower part of the South Island of New Zealand (near Dunedin and Invercargill), while those that were collected after a longer time off feed were collected in the lower part of the North Island (in Palmerston North). This may have some impact on conclusions from the Grass diet, with pasture compositions and quality likely differing between the two locations. Although we do expect some differences in metagenome profiles from samples collected a short or a long time off feed [19], this may be an explanation for the extent of the differences we observe between these Groups (Figs. 1A, 3, 4A, 5A, 6A and 7A).

Our study has shown that the major influences on microbial abundances are similar for the range of microbes represented in the Hungate1000 Collection, however, there are some other, typically more minor influences, that are not so consistent. For example, in our study, the Cohort effect captures the major environmental parameters (diet, age, time off feed, and Cohort). Therefore, for prediction purposes, a Cohort adjustment will adjust those taxa for the major effects on abundance, while avoiding over-adjustment, which would likely result in a loss of important signal. However, when using RE-RRS metagenome profiles to evaluate individual taxa, it may be important to include systematic and biological effects such as Library, Batch, Shelf, Well, BRR, AOD and BDEV in the model.

### Relationships between samples from different Groups

We employed a network-based approach to explore the key drivers of similarities between microbial profiles using four different methods of microbial profiling: RB (Fig. 4), RF with tags generated on the full set of samples (Fig. 5), RF with tags assigned taxonomy at the genus level (Fig. 6), and RF considering the set of tags that were present in all of the eight Groups (Fig. 7). In all cases, samples primarily clustered by differences in diet (Figs. 4A, 5A, 6A and 7A), however, within a diet, there was further clustering based on Group and there was no obvious clustering based on methane selection line (Figs. 4C, 5C, 6C and 7C). The RF approach was shown to be more sensitive at capturing relationships between samples, for example, the RF approach (Fig. 5) had stronger relationships between samples within each Group (tighter clusters) and weaker relationships between samples in different Groups (clusters are further apart from each other) than the RB approach (Fig. 4).

After Cohort adjustment, network diagrams from the RB profiles (Fig. 4) did not show clustering based on Group, although network diagrams from the RF profiles (Fig. 5) still showed some structure based on Group. Because the RF profiles were generated from a set of tags that were present across all samples, some tags would be unique to a particular Group (Fig. 1). Therefore, the clustering that remained after Cohort adjustment was likely due to some of the tags being fixed (e.g., abundance of zero) in some Groups and varying in others, thereby being relatively unaffected by the Cohort adjustment approach we have used in this study. Based on our approach of normalizing within Cohort, if there were some Cohorts or Groups where several tags were not captured, these tags would be reducing the relationship coefficient (correlation) between samples from different Groups compared to a profiling approach that did not include these tags. This is supported by network diagrams from profiles with tags assigned at genus level (Fig. 6) and profiles with tags present in the tag set of all Groups (Fig. 7), where the unadjusted network diagrams showed the different Groups appearing closer together than when using the full RF profiles (Fig. 5).

Sheep with extremely high or low methane emissions have been shown to have different rumen microbiomes [2], therefore it was unexpected that samples did not appear to cluster by methane selection line in the unadjusted network diagrams (Figs. 4C, 5C, 6C and 7C). However, in Kittelmann et al. [2], the sheep were fed a single diet and were from a single Cohort, therefore there were fewer environmental influences on the microbial profiles than in the present study. In the present dataset there was the presence of a signal of the large environmental effects of Group and Cohort and, after adjusting profiles for these effects, samples showed clear clustering based on methane selection line (Figs. 4D, 5D, 6D and 7D). The PERMANOVA analysis of relative abundances of RB and RF metagenome profiles showed that although the methane selection line signal was not observed in the network diagrams, there was still clear clustering based on methane selection line, even though the Group effects are much stronger. This shows that environmental factors may mask important biological signals [11] but biological signals are still present within the profiles, and can be revealed by using Cohort-adjusted metagenome profiles from RE-RRS. Additionally, the clear separation of the selection lines observed after Cohort-adjustment suggests that these profiles are likely to be reasonable predictors of methane emissions.

### Taxon abundances

Our RB approach used the Hungate1000 Collection [13] to assign sequences to taxa at the genus level. The Hungate1000 Collection contains genome assemblies of many cultured microbes from a variety of ruminant species, and these genome assemblies represent every cultivated rumen-associated archaeal and bacterial family. However, there are numerous microbes that are currently unable to be cultured and therefore RB microbial profiles based on the Hungate1000 Collection will be missing important microbes.

Although the RF approach, by definition, would not typically involve the taxonomic assignment of tags, we felt it was valuable for the purposes of this study to explore the extended taxonomic range that the RF approach was able to capture compared to the RB approach. Table 4 showed that the proportion of tags that were able to be assigned to a particular taxonomic level was consistent across the different tag sets in our study, and the largest change in the proportion of tags assigned was approximately 10% between the species and genus levels, with the difference between genus and domain assignment rates being less than 10%. This is likely due to the optimization of the RF approach to assign taxonomy at the genus level, as described by Hess et al. [12]. However, it also indicates that some tags will be representative of multiple genera within a family, while others may be representative of intra-species variation. The highest assignment rates were from the tag set where tags had to be present in all of the eight Groups in our study. Assignment rates were similar between Groups and even the proportion of tags with taxonomy assigned was consistent between the NZ and AUS tag sets at all taxonomic levels. This is likely because taxa that are present across a range of diets and environments are more likely to have genome assemblies, and therefore appear in our databases, than those that have a more specific niche.

Taxonomic assignment of tags (Fig. 2) showed reasonably similar results to those reported by Hess et al. [12], although a smaller proportion of reads was unassigned (58% compared to 71%). The major taxa identified within each domain were also consistent with those reported by Hess et al. [12], although these generally had a slightly different proportion of tags assigned to each of them. These observations were most likely caused by the reads in our current study needing to be present in 25% of samples from a range of diverse environments and diets, compared to the single environment and diet the sheep from Hess et al. [12] were exposed to. Therefore, it is important to carefully consider the set of samples used to generate the tag set when using the RF approach to avoid removing informative sequences prior to downstream analysis.

Taxon abundances were reasonably consistent between the RB and RF approaches (Fig. 3), however there was some rearrangement in abundance rankings of some families. *Prevotellaceae* was the most abundant rumen microbe in all Groups, consistent with other studies in ruminants [2, 11]. The RF approach resulted in a larger proportion of the metagenome profile assigned to the “Other” category, which contained families with sequences present at less than 1% of bacterial or archaeal sequences, showing that it was capturing a broader range of rare taxa. Additional genome assemblies in the GenBank database can cause a reordering of the abundance of families in two ways: firstly, sequences that were originally unassigned based on the Hungate1000 Collection may be assigned under the RF approach, causing new families to emerge in the RF plot (Fig. 3B) compared to the RB plot (Fig. 3A); secondly, sequences that were assigned to one taxon using the Hungate1000 Collection may have a better match with a genome from a different family in the GenBank database, which may result in the reordering of affected taxa. Other taxa may be captured by RB profiles but not RF profiles: *Streptococcaceae* is an example of a family that was captured at high abundance (> 1%) in the RB approach but not in the RF approach. This was because *Streptococcaceae* had low abundance in all but the Australian samples, and so many sequences originating from this family likely did not reach the 25% prevalence threshold to be considered tags when the tag set was generated across all samples.

### Heritability and repeatability estimates

Repeatability of taxon abundance is a measurement of how consistent the abundance of a particular taxon is between different samples taken on the same individual. Heritability of taxon abundance is a measure of the extent to which the abundance of a particular taxon is more similar in samples from related sheep than unrelated sheep. Heritability is a component of repeatability, and so repeatability estimates are equal to or higher than heritability estimates. Host genetics have been shown to play an important role in gut microbial abundances in a range of species, e.g., humans [21], mice [22], and cattle [9, 10]. Taxa that are heritable or repeatable provide a valuable source of information for selection of individuals, for either genetic selection, or animal management purposes, respectively.

Heritability and repeatability of taxon abundances were estimated based on the three datasets: sheep fed grass, sheep fed lucerne pellets and all New Zealand samples (Figs. 8 and 9, and Additional File 4). Heritability and repeatability estimates were not calculated for the Australian samples because the dataset was small and there was only one rumen sample for each individual, encumbering the ability to obtain reliable estimates. Correlations between sheep fed grass and all New Zealand samples were highest because the samples from sheep fed grass made up the majority of the full New Zealand dataset. Heritability and repeatability estimates from the lucerne pellet diet tended to be higher than those from the grass dataset. Two reasons for these higher heritability estimates may be: (1) the lucerne pellet diet is a more consistent diet than the fresh ryegrass-based pasture, which changes in composition from day-to-day, with changing seasons and from different regions of New Zealand; and (2) the samples collected from sheep on the lucerne pellet diet were all collected from lambs, whereas samples collected from sheep on the grass diet were collected from both lambs and adults. Increased variation in diet and age may increase the environmental variation of taxon abundances, resulting in lower heritability estimates. The correlation of heritability and repeatability estimates between the three datasets were moderate to high, suggesting that the host control of the rumen metagenome is reasonably consistent across these different environments.

*Quinella* and *Succiniclasticum* were two genera that had high heritability and repeatability estimates from both the RB and the RF profiles (Figs. 8 and 9 and Additional File 4). A number of genera that were identified as highly heritable or repeatable in the RF profiles are likely to be mis-assigned. Steinegger and Salzberg [23] identified over two million contaminated genome assemblies in GenBank, and De Simone et al. [24] discussed how easily bacterial (and other) sequences can contaminate genomes and suggested ways to reduce the likelihood of this occurring. *Buceros* is a genus of hornbill and had one of the higher heritability estimates and was in the top 40% of most common genera, indicating that a microbe that is closely related to a rumen microbe is likely to have been unintentionally inserted into a *Buceros* genome assembly. Another unexpected genus that was identified as heritable and repeatable from the RF profiles was *Apis*, representing honeybees, and an explanation for this is not clear. It is feasible that a honeybee may have been present in the rumen of sheep if the sheep ingested one while foraging; however, in this situation the chance that the abundance of *Apis* sequence in the rumen is heritable is reasonably low. *Apis* abundance could be heritable if it was related to foraging behavior (e.g., some sheep may preferentially graze clover which may be more likely to have honeybees in it), however the heritability estimate of 0.21 ± 0.04 in the Grass dataset is reasonably high. It is also possible that the honeybee genome contains contaminated sequence from the environment, given that *Apis* was significantly heritable in both the Grass and Lucerne Pellet datasets.

### Reference database design

The ability to assign taxonomy to reads or tags is limited by the reference database that is being used and gaps in current databases mean that informative taxa are likely not being captured within our current metagenome profiles. Improvement in the reference databases used to assign taxonomy to reads or to cluster tags would be valuable. This would require a concerted effort, since high confidence taxonomic assignment of sequences can be time consuming and expensive, especially on a large scale. The Hungate1000 Collection is a database of high-quality genome assemblies from cultured microbes, where the taxonomy is reliably known; however, it is from a limited number of taxa. In comparison, the GenBank database contains a much larger taxonomic range, but the taxonomy and quality is known with less confidence [23]. There are cases where contamination in genome assemblies can lead to incorrect taxonomic assignments [23, 24], and in the case of our approach, this can lead to either incorrectly assigned or unassigned tags, depending on whether there were similar sequences with the correct taxonomic assignment in the database. Although the taxonomic range of the GenBank database is larger than the Hungate1000 Collection, it is by no means complete and uncultured microbes are still underrepresented [25]. An example of this is the appearance of taxonomic groups such as *Halobacteria* (Fig. 2), which is indicative that a group of methanogens is missing from the GenBank database, as *Halobacteria* are not found in the rumen (they are found in high-salt waters, e.g., the Dead Sea) but are close relatives of some known methanogens.

The additional families that were identified in the RF metagenome profiles but not in the RB metagenome profiles (Fig. 3) provide a source of additional genome assemblies that may be valuable to include in the reference database for the RB approach. In particular, genome assemblies from the *Rikenellaceae* family, the RC9 gut group, have been poorly classified but are thought to be able to degrade cellulose and play an important role in rumen fermentation [11, 13], and therefore may play an important role in traits such as methane emissions and feed efficiency. The abundance of the *Rikenellaceae* RC9 gut group has previously been shown to be negatively associated with average daily gain in sheep fed a concentrate diet [26] and abundance tends to be higher on low-starch or forage-based diets in a variety of ruminant species [27–30]. The RC9 gut group is present within the full Hungate1000 Collection, however due to our approach of assigning taxonomy at the genus level, the RC9 genomes are not currently part of the set of assemblies considered in the RB approach as their taxonomy is not known to the genus level.

Metagenome-assembled genomes (MAGs) are putative genome assemblies that are obtained from whole-metagenome sequencing of one or more metagenome samples. Catalogs of MAGs have been generated in a wide range of environments, including various sites on the human body [31], cattle rumens [32] and thermal hot pools [33]; and have been shown to vastly improve the number of mapped reads compared to traditional databases of genome assemblies, such as the Hungate1000 Database [13] for rumen samples. One major reason for this improvement is that MAG catalogs typically capture uncultured microbes, which are largely unrepresented in most genome assembly databases [25]. Although the capture of novel genome assemblies improves the number of reads mapped, many MAGs don’t have a taxonomic assignment, therefore it may be difficult to bin reads if they map to multiple MAGs. Another complication with MAGs is that a given MAG may be a chimera of multiple true genomes, and clustering tags or reads based on mapping to chimeric genome assemblies may result in the loss of signal if that tag is associated with a trait of interest. Stewart et al. [32] were able to use a MAG catalog generated from cattle rumen samples to improve the proportion of reads mapped from sheep rumen samples from ~ 15% to ~ 55%. The sheep samples used in Stewart et al. [32] were from Shi et al. [34] and included individuals selected for high and low methane emissions. Shi et al. [34] found no significant differences in gene abundance between high and low methane individuals, while Stewart et al. [32] were able to identify significant differences in abundance of particular taxa and MAGs between the high and low methane animals. This highlights the potential of MAG catalogs for improving the amount of information that can be used from RE-RRS of rumen metagenome samples by increasing the number of reads or tags that can be assigned. This may improve the ability to associate rumen metagenomic profiles with traits of interest.

### Profiling approaches for the future

We explored two different approaches for metagenome profiling: a reference-based (RB) and a reference-free (RF) approach. Metagenome profiles from the RB approach are currently restricted to capturing organisms that are present in the Hungate1000 Collection [13] plus four *Quinella* genomes, and are therefore limited to culturable microbes. Metagenome profiles from the RF approach are not restricted in this way, and we have shown that this approach captures a much broader taxonomic range (Figs. 2 and 3). However, Cohort adjustment of the RF profiles did not remove biological effects of Group to the same extent as was seen in the RB profiles (Figs. 4 and 5). This is likely due to several tags being absent for some Cohorts (i.e., zero counts for a tag for all individuals in a Cohort), resulting in little to no impact of Cohort adjustment for those tags. There are numerous filtering and clustering methods that could be used to overcome this issue. We explored two approaches: (1) clustering tags based on taxonomic assignment at the genus level and (2) filtering tags such that only tags that were identified in all of the eight Groups were included, i.e., those that were prevalent across a wide range of environments. The profiles based on clustering (Fig. 6) and filtering (Fig. 7), RF tags only account for 12.1 ± 2.3% and 8.1 ± 1.8% of reads, respectively, compared to 29.2 ± 3.8% for the full RF profiles and 20.8 ± 3.5% for the RB profiles. However, the clustered (Fig. 6D) and filtered (Fig. 7D) metagenome profiles actually show clearer clustering by methane selection line than the full RF (Fig. 5D) or the RB profiles (Fig. 4D), indicating that these profiles may be more robust. These results show that RF metagenome profiles can be filtered or clustered to reduce dimensionality without a loss of meaningful biological signal.

A heritable genetic component to rumen metagenome composition provides an avenue for sustained differences in rumen metagenome composition across generations and, consequently, traits influenced by the rumen metagenome (e.g., feed efficiency and greenhouse gas emissions). Repeatability represents the robustness of the abundance of taxa across time and provides an opportunity to use metagenome profiles to select individuals who will have reduced methane emissions across their lifetime. We have shown that several genera from both RB and RF approaches are heritable and repeatable (Figs. 8 and 9), indicating that selection on the rumen metagenome will be sustained across an individual’s lifetime, as well as across generations. Filtering metagenome profiles to retain only those taxa or tags that are heritable is one approach that would reduce the metagenome profile to the subset that is able to produce sustained differences in metagenome profiles across generations and may reduce the noise in the RF metagenome profiles.

A Metagenome Wide Association Study (MWAS) could be performed to identify associations between the abundance of each genus or tag and a trait of interest (e.g., methane emissions). This approach could provide an additional source of information for filtering tags or genera to generate a metagenome profile that is designed specifically for predicting a trait of interest. Care would need to be taken when using this approach to ensure the dataset is large enough to perform this type of analyses (e.g., thousands of individuals with metagenome profiles and phenotypes for the trait of interest) and selecting only the taxa that are significantly associated with the trait of interest could result in the omission of other taxa that are associated with the trait.

The different approaches for generating microbial or metagenome profiles in this study show the flexibility of RE-RRS for a variety of different studies. The RB approach was based on a database of high-quality microbial genome assemblies where taxonomies were reliably known, however, while taxonomic assignments from this approach were known with high confidence, they only covered a limited taxonomic range. Future studies could use a more comprehensive database, including GenBank and MAG databases to capture a much broader taxonomic range. However, using a large database such as this would drastically increase computation time when obtaining metagenome profiles on a large number of samples, as was done in our study. The RF approach was shown to capture a greater proportion of sequences than our RB approach without the additional step of taxonomic assignment. Taking such a black box approach is suitable for use in an MRM for trait prediction, but (on its own) may limit the biological insight that could be gained if taxonomy was known. We assigned taxonomy to RF tags using the sheep genome, Hungate1000 Collection and GenBank to investigate the taxonomic range. While this approach could allow biological insights beyond the standard RF approach, we found that there were fewer reads assigned when clustering tags based on taxonomic assignment at the genus level than even the RB approach, showing that clustering RF tags may miss some meaningful biological signal. The method to get the highest assignment rates is likely to be the RB approach using a database that contains the sheep genome, Hungate1000 Collection, GenBank, and rumen-specific MAG databases; however, the computational requirements for this approach would be extreme, and not consistent with a high-throughput metagenome profiling approach. The most suitable approach for bioinformatic processing of RE-RRS reads into metagenome profiles is therefore clearly dependent on the purpose of the analysis to be undertaken.

### Towards integrating metagenome profiles in trait predictions

The integration of rumen metagenome profiles into predictions of economically and environmentally important traits has been investigated in several species and traits [5, 6, 10, 35, 36], however research published to date has used relatively low numbers of samples due to the lack of availability of a low-cost, high-throughput metagenome profiling approach, such as RE-RRS. Many of these studies have generated a microbial relationship matrix (i.e., MRM), calculated in a variety of ways, to represent the relationships between samples, followed by a microbial BLUP (i.e., MBLUP) approach [5, 6]. As discussed by Ross and Hayes [37], these studies have shown promise for using microbial profiles to predict individual performance. While not addressed directly in our study, clustering of individuals by methane selection line suggests an ability to predict environmentally important traits in sheep using this information. If metagenome profiles can be used as proxies for traits such as feed efficiency or methane emissions, then the need to collect these expensive and time-consuming phenotypes on large numbers of animals is greatly diminished.

The ability of a method to predict performance is dependent on the information content contained within the predictor. The RF approach captured a greater proportion of reads per sample than the RB approach, consistent with findings from Hess et al. [12], even when RF tags were generated on the full dataset (All Samples; Table 2 and Additional File 2). Unlike 16S rRNA gene sequencing or using a bacteria-oriented reference database (i.e., the Hungate1000 Collection), information on other organisms within the rumen (e.g., parasites) are additionally captured using the RF approach (Fig. 2). The RF approach was shown to be a more powerful approach to generating metagenome profiles than the RB approach, with samples from sheep fed the same diet clustering more closely together than samples from sheep fed different diets in the network diagrams (Figs. 4A and 5A). This indicates that metagenome profiles from our RF approach may be more successful at predicting traits of interest than RB metagenome profiles. Hess et al. [38] explored the use of different metagenome profiles from this study for prediction of a variety of economically and environmentally important traits. The large dataset presented in our paper provides a valuable resource for developing and benchmarking different approaches to integrating metagenomic profiles into trait predictions, and this is the current focus of ongoing research.

## Conclusions

RE-RRS is a high-throughput approach that can be used to cost-effectively obtain thousands of rumen metagenome profiles. We have shown that the variation in rumen metagenome profiles generated in sheep using RE-RRS is controlled by both the host (i.e., genetics, age) and its environment (i.e., diet, Cohort). After normalization of relative abundances within Cohort, a methane yield selection line signal was detected. Using an RB or RF approach to generate rumen microbial profiles resulted in similar relationships between samples, however, the RF profiles showed clearer separation between Groups, overcoming the limitations of an incomplete reference database. Although a RF approach provides more information, reduction of noise through filtering or clustering allowed a stronger signal to be realized. The influence of genetics on rumen metagenome profiles suggests the potential to select on the rumen microbiome to make sustained progress in other related traits, such as methane emissions. These results are promising for the ability to accurately predict methane emissions across diet, age, and generation, but also highlight the importance of adjusting Cohort effects out of metagenome profiles prior to prediction.

## Methods

The animal experiments conducted in New Zealand adhered to the guidelines of the 1999 New Zealand Animal Welfare Act and AgResearch Code of Ethical Conduct. New Zealand trials of the current study were approved by the AgResearch Invermay (Mosgiel, NZ) and AgResearch Grasslands (Palmerston North, NZ) Animal Ethics committees under approval numbers 12414, 13,081, 13,419, 13,563, 13,742, 13,892, 14,055, 14,066, and 14,221. Australian animal experiments were approved by the University of New England Animal Ethics Committee, under AEC 15–021.

### Rumen sample collection

Rumen samples were collected in New Zealand and Australia on a total of 1,708 sheep. Most New Zealand sheep had multiple samples collected on them, while only one sample was collected from the Australian sheep. Rumen samples on New Zealand sheep were collected under multiple different conditions, including diet, age, time off feed before the sample was collected, and whether they were fed ad libitum or at a restricted feeding level. New Zealand samples were classified into seven different Groups along with an additional Group for the Australian sheep, as outlined in Table 1. Further details about the collection and processing of the sheep samples are described below.

#### New Zealand animals and rumen sample collection

Sheep rumen contents (~ 30 mL) were sampled via stomach intubation followed by freezing at − 20 °C. Samples were collected from 1,200 dual-purpose composite ewes, with 3.3 ± 1.4 samples collected per sheep, totaling 3,971 New Zealand samples (Table 1). These samples were collected as part of a variety of feed intake [39] and methane [18, 40, 41] measurement trials at the Grasslands (Palmerston North), Invermay (Mosgiel) and Woodlands research farms of AgResearch in New Zealand. Across the trials, the animals were sampled when they were on three different diets: lucerne (alfalfa) pellets, maintenance (low metabolizable energy) pellets, or New Zealand ryegrass-based pasture; at two different life stages: lambs (< 15 months) and adults (> 15 months); and two different times off feed: a short time off feed (2–4 h) or a long time off feed (15–16 h). Animals were born between 2014 and 2016 and were from three New Zealand sheep flocks: one AgResearch flock (Flock 2638), one selection line flock containing sheep selected for high or low methane yield (Flock 3633) [16, 17], and one Central Progeny Test flock (Flock 4640). There were 118 sires represented across the 1,200 sheep and each sire had 10 ± 5 offspring. Further details for each sample can be found in Additional File 1.

#### Australian animals and rumen sample collection

Australian rumen samples were collected from sheep that were part of a methane experiment as described by Robinson et al. [42]. Animals were fed a diet of equal parts chaffed alfalfa and cereal hay (Manuka Feeds, Quirindi, NSW) at 1.5 or 1.6 maintenance requirements, calculated from the weight of the animals prior to transport. Ewes were measured twice in portable accumulation chambers (PAC) and twice in respiration chambers (RC) across seven trials run in Armidale, New South Wales, Australia, between April 2015 and March 2016. Rumen samples were collected by stomach intubation approximately 10 min after the second PAC measurement was completed, when the sheep had been off feed for approximately 2 h. Sheep used in that study consisted of Information Nucleus Flock follower ewes, born between August 2007 and October 2013. These ewes are primarily Merinos and are representative of, and have genetic links with, the genotypes currently used in the Australian sheep industry [43, 44]. The 488 sheep that had rumen samples came from 176 sires [45]. Additional details for each sample can be found in Additional File 1.

### Sample processing

#### New Zealand samples: Freeze drying

The New Zealand samples were freeze dried at AgResearch Ltd.’s Invermay campus. Samples were randomly allocated into Batches of ~ 93 samples based on sampling Cohort, as they became available for freeze drying (i.e., Cohorts were spread across Batches). Two Batches of samples were freeze dried simultaneously. Samples had their lids removed and were placed on stainless steel trays in a − 20 °C freezer for approximately four hours. Samples from each Batch were randomly allocated across the five shelves of a CHRIST Gamma 1–16 LSC plus freeze dryer (CHRIST, Osterode am Harz, Germany). In the first 10 min, the freeze dryer reduced the pressure from 1000 mbar to 0.5 mbar, this pressure was held for the next six hours, before dropping to 0.3 mbar over the next 96 h and held at that pressure until they were removed from the freeze dryer seven days after they were put in. After freeze drying, the lids were immediately replaced, and the samples returned to the − 20 °C freezer.

#### Australian samples: Freeze drying

The 508 Australian samples were freeze dried at the University of New England using 5 different freeze-drying approaches. These approaches are outlined in Additional File 5, along with a comparison of the different approaches and justification for inclusion of all samples in this manuscript. Two freeze driers were used for the Australian samples: a CHRIST Alpha 1–4 LD plus freeze drier (CHRIST, Osterode am Harz, Germany) and a Dynavac FD-PILOT 7–12 freeze drier (Dynapumps, Seven Hills, NSW, Australia).

#### Australian samples: Transport

To transport freeze dried samples to New Zealand, small groups of samples were taken from the freezer and placed on ice. Individual samples were opened, then the contents were broken up and stirred using a fresh pipette tip for each sample. The whole dried sample was tipped into a 5 mL tube and the lid screwed on. The label from the initial sample was peeled off and placed on the new 5 mL tube. Samples were then returned to the − 20°C freezer. Samples were shipped on dry ice from Armidale, NSW, Australia to Mosgiel, New Zealand and arrived frozen with no indication they had defrosted during shipping.

#### All samples: grinding

Freeze dried samples were ground into a powder to homogenize the sample so that the subsample that DNA was extracted from was representative of the full sample. Samples were ground using a Magic Bullet kitchen blender (NutriBullet New Zealand, Auckland, New Zealand) with a custom-made cup. Samples from Australia and those from the grass diet were ground for 10 s continuously, whereas samples from the pellet diet required 30–40 s of grinding, in 5–10 s pulses, to appropriately homogenize. A small number of samples needed an additional 5 s of grinding to homogenize. New Zealand samples that were from the same Batch and freeze dried on the same Shelf were ground in the same session. Australian samples that were freeze dried using the same approach were in the same Batch for grinding; however, the largest set was too large to grind in one session, so the samples were processed in smaller Batches, ensuring that samples from the same trial were in the same Batch for grinding.

### DNA extraction and sequencing

DNA was extracted from rumen samples using a combined bead-beating, phenol and column purification protocol using the QIAquick 96 PCR purification kit (Qiagen, Hilden, Germany), as described in Text S1 of Kittelmann et al. [2], to provide high quality nucleic acids for RE-RRS. RE-RRS was carried out as described by Hess et al. [12]. Briefly, after digestion of DNA by the restriction enzyme *Pst*I, barcodes were ligated to link sequences to samples, as described by Elshire et al. [46] with modifications as outlined in Dodds et al. [47]. Four 96-well plates (one plate per freeze-dried batch) were grouped into each library and PCR amplified with 18 cycles. Assignment of samples to wells within a plate was random. Amplified sequences between 193 and 318 bp were selected using a Pippin Prep (SAGE Science, Beverly, Massachusetts, USA) and each library was run on a single lane of a flow cell on an Illumina HiSeq2500 machine (San Diego, California, USA), generating 101 bp single end reads using version 4 chemistry. Sequencing ~ 376 samples per lane was shown to be a very conservative sequencing depth by Hess et al. [12], who found that there could be 10 times this number before there was any loss in metagenome diversity.

### Metagenome profiling

Demultiplexing and trimming were carried out using GBSX [48] and Cutadapt [49]. GBSX was run using default parameters except no mismatches were allowed in the barcode or cut site. Cutadapt was run with a Phred quality score threshold of 20 and a minimum length of 40 base pairs. All samples with fewer than 100,000 reads after quality control were considered failed samples. Failed samples were re-sequenced in another Library and if they failed again the sample was removed from future analyses. After this quality control, sequences were run through both the reference-based (RB) and the reference-free (RF) pipelines, briefly described below, with further details in Hess et al. [12]. Both pipelines produce a table of counts with one row per sample and one column per genus (RB) or tag (RF).

#### Reference-based pipeline

The RB pipeline used nucleotide BLAST (task = blastn, word size = 16, e-value = 0.01) in BLAST v2.2.28 + [50] to compare sequences against a reference database. Taxonomy of sequences was assigned using an R implementation of the algorithm from MEGAN [51] with default parameters. The microbiome profile for each sample was the number of sequences assigned at the genus level for each of the 61 genera represented in the reference database.

The reference database contained four *Quinella* genome assemblies [14] as well as a filtered version of the Hungate1000 Collection of rumen microbial genome assemblies [13]. Filtering of the Hungate1000 Collection involved the removal of genome assemblies where taxonomy was not known to the genus level (e.g., taxonomy only known to family or order level), to maximize the number of reads that could be assigned at the genus level using the MEGAN algorithm.

#### Reference-free pipeline

The RF pipeline generated a set of “tags”, which are non-redundant 65 base pair long sequences that commence at the initial cut site and are observed in at least 25% of samples. The metagenome profile contains the number of times each tag is observed in each sample. Tags were generated for the set of all samples, as well as separately for each of the Groups in Table 1 using an in-house Unix script. Any sequences that were shorter than 65 bp were ignored, as they did not match the full 65 bp tag.

#### Filtering and clustering of reference-free tags

Given the large number of tags that were generated from the RF pipeline, we investigated one method for filtering tags and another method for clustering tags. The approach to filter tags retained only the tags that were identified in all eight Groups. The second approach was to cluster the set of tags that was generated on all samples (i.e., the “All” set) based on taxonomy. The first step in assigning taxonomy to tags was to compare the tags against the sheep genome (OAR 3.1) using bwa mem [52] with default parameters, and considering those tags with a flag of 0 or 16 (i.e., uniquely mapping in the forward or reverse direction) to be tags that come from sheep. The second step to assigning taxonomy to tags was to use nucleotide BLAST (task = blastn, e-value = 0.01) in BLAST v2.2.28 + [50] to compare tags that didn’t align to the sheep genome against our reference database (used for the RB approach) and GenBank [15]. Taxonomy was assigned to tags using the MEGAN algorithm written in R, as described in the RB section, however, only hits that had a bitscore equal to the top bitscore for that tag were considered. Tag taxonomies were considered at various taxonomic levels (e.g., domain, genus), and read counts of tags assigned to the same taxonomic group at the relevant level were summed together, with those not assigned at the given taxonomic level ignored.

#### Adjustment of metagenome profiles

Metagenome profiles from the RB and RF pipelines were transformed into relative abundance matrices by dividing each count in the count matrix by the row sum (i.e., the number of reads assigned for that sample). Transformation to log_10_ proportions was performed by adding one to all counts in the count matrix and dividing by the new row sum (i.e., the number of reads assigned for that sample plus the number of taxa) then taking the log with base 10. A “log_10_ normalized” metagenome profile was generated by normalizing each column in the log_10_ proportion matrix, such that each column had a mean of zero and standard deviation of one. In addition to this, a “Cohort-adjusted” metagenome profile was generated whereby each column of the unadjusted metagenome profile was log_10_ normalized within Cohort such that within each column (i.e., genus or tag), each Cohort had a mean of zero and standard deviation of one. Normalization in this way gives each microbe the same weighting in the profile regardless of their abundance, which avoids drowning out signals from lowly abundant but potentially biologically important microbes. It also reduces any biases between genera/tags, such as genomes that have a larger or smaller proportion of their genome captured by RE-RRS because each genus/tag abundance is reported relative to the average abundance of that genus/tag.

### Biological and systematic effects

Models were run in ASReml 4.1 [53] to evaluate systematic and biological effects on the number of reads, proportion of reads that were assigned using the RB and RF approaches, and the relative abundance of each genus from the RB log_10_ normalized metagenome profiles. The systematic and biological effects on the relative abundances of each tag in the RF metagenome profiles were not evaluated because there were typically over 200,000 tags for each of these datasets. Models were run using the New Zealand samples only, where model equations were of the form:

1 $$y = \mu +\mathrm{ Group }+\mathrm{ Cohort }+\mathrm{ Library }+\mathrm{ BatchShelf }+\mathrm{ Well }+\mathrm{ BRR }+\mathrm{ AOD }+\mathrm{ BDEV }+ PE + e$$

where *y* is the dependent variable: total number of reads, proportion of reads assigned, or taxon relative abundance; *µ* is the overall mean; Group is the fixed class effect of Group (Table 1); Cohort is the fixed class effect which included sampling cohort and flock (all individuals from a Cohort are in the same Group); Library is the fixed class effect of the library the sample was assigned to for sequencing; BatchShelf is the combined fixed class effect of the Batch and Shelf the sample was on for freeze drying; Well is the fixed class effect of the position on the plate (well) for DNA extraction and sequencing; BRR is the fixed class effect of birth and rearing rank, which is the combination of the number of lambs born to the individual’s mother at the time of their birth and the number of those lambs raised to weaning; AOD is the fixed class effect of age of dam at the time the individual was born (1, 2, 3 +); BDEV is the fixed covariate of the deviation of the individual’s birth date from its contemporary group average; *PE* is the random animal permanent environmental effect with variance structure **P** ${\sigma }_{PE}^{2}$ where **P** is a matrix linking samples from the same individual; and e is the residual with variance structure I ${\sigma }_{e}^{2}$.

### Comparison of tags

Tags were generated from the full set of samples, as well as for each Group. To determine which Groups have more similar tag sets, a dendrogram was generated in R using the hclust function with method = “average”, based on a distance matrix that was computed as the proportion of tags that differ between Groups. Venn diagrams were generated using the VennDiagram package in R to compare the tags that were unique to a Group and those that were in common between Groups. These parameters are displayed in terms of the number of tags as well as the percentage of tags. Three Venn diagrams were generated: one for the sets consisting of sheep fed a grass diet (GAS, GAL, GLL and GLS), one consisting of sheep fed a pellet diet (LLL, LLS and MAS) and one consisting of sheep from New Zealand (NZ) and Australia (AUS).

### Relative abundance graphs

Relative abundances of bacterial taxa and archaea were compared between Groups using the RB profiles and the RF profiles where the tags had been clustered based on taxonomic assignment. These graphs were generated in R using the barplot function and were visualized at the taxonomic level of family.

### Network diagrams

Network diagrams were generated using NetView [54] in R using default values with the Walktrap algorithm and k parameter set to 150 to visualize relationships between samples. The network diagrams were generated using correlation matrices on both the log_10_ normalized and Cohort-adjusted metagenome profiles. Each correlation matrix was generated using the cor function in R which resulted in an *n* × *n* matrix, where *n* is the number of samples. These network diagrams were generated using all samples. Network diagrams were coloured based on Group and methane selection line (with individuals from Flocks A and C coloured grey).

### PERMANOVA Analysis

A PERMANOVA analysis was run on RB and RF profiles to identify whether profiles were significantly different between Groups, and between samples from different methane selection lines. Relative abundance profiles were analysed, with all samples used to analyze the effect of Group, and only samples from the methane selection lines used to analyze the effect of selection line. When testing Group effects, only Group was fitted in the model, but when testing selection line effects, both Group and selection line were fitted in the model. The analysis was run using the adonis2 function in the vegan package [55] in R with permutations = 999 and method = “bray”.

### Heritability and repeatability

#### Animal Genotyping

The New Zealand animals were genotyped using Illumina beadchip assays, ranging in density from 5,000 to over 600,000 markers, with low density panels being imputed up to the greatest density panel using a large reference population of New Zealand sheep. The genotyping panels (number of animals) used included the AgR Custom 5k LD (*n* = 17,631), AgR Custom 6k LD (*n* = 8,798), ISGC 15K (*n* = 25,831), AgR Custom 18k LD v2A1 (*n* = 24,912) and AgR Custom 18k LD v2C1 (*n* = 4,695), and the ISGC Ovine HD (*n* = 22,802) chips. SNPs were filtered such that (a) both probes uniquely mapped to the OAR 3.1 genome, (b) one probe mapped with zero mismatches, (c) no indels were found, and (d) both probes were in the same orientation and position with exactly one mismatch (the target SNP). Subsequently, each genotype panel was imputed from its respective density to the 568,142 SNPs retained on the ISGC Ovine HD panel using Beagle v5.1 [56] with default settings, except for effective population size which was set to 500. The phased and imputed set was then reduced to only include the 1,200 sheep from New Zealand used in this study (AgR Custom 5k LD: *n* = 124, AgR Custom 6k LD: *n* = 9, ISGC 15K: *n* = 613, AgR Custom 18k LD v2A1: *n* = 24, AgR Custom 18k LD v2C1: *n* = 3, ISGC Ovine HD: *n* = 430). A genomic relationship matrix was calculated from all 568,142 SNPs using the first method of van Raden [57] and can be found in Additional File 6. No genotypes were required for the Australian samples as the dataset was small to calculate reliable heritability estimates.

#### Analysis

Heritability and repeatability were estimated for each genus in the reference database for the RB profile and for each genus in the RF profile where tags were clustered based on genus level taxonomic assignment. These estimates were computed using three different sets of samples that consisted of (a) all the samples where sheep were fed grass (GAS + GLS + GAL + GLL), (b) all the samples were sheep were fed lucerne pellets (LLS + LLL) and (c) all the New Zealand samples. Each set had rumen samples from slightly more than 1,000 individuals and the number of samples varied between 1,361 and 3,971 across the three Groups. Estimates were computed using Cohort-adjusted metagenome profiles and were run in ASReml 4.1 [53] using the model:

1 $$y =\upmu + Animal + PE + e,$$

where *y* is the Cohort-adjusted log_10_ proportion of each genus in the profile (i.e., each column of the Cohort-adjusted metagenome profile); *µ* is the overall mean; *Animal* is the random animal genetic effect with variance structure **G** ${\sigma }_{g}^{2}$ where **G** is the genomic relationship matrix; *PE* is the random animal permanent environmental with variance structure **P** ${\sigma }_{PE}^{2}$ and *e* is the residual with variance structure **I** ${\sigma }_{e}^{2}$.

The total variance was calculated as the sum of the variances due to the animal genetic effects, the animal permanent environmental effects and the residual effects (i.e., total variance = ${\sigma }_{T}^{2}= {{\sigma }_{g}^{2}+\sigma }_{PE}^{2}+{\sigma }_{e}^{2}$). The heritability was estimated as the variance due to the animal genetic effect divided by the total variance (i.e., ${\sigma }_{g}^{2}/{\sigma }_{T}^{2}$) and the repeatability was calculated as the sum of the animal effect and permanent environmental effect variances divided by the total variance (i.e., $({\sigma }_{g}^{2}+{\sigma }_{PE}^{2})/{\sigma }_{T}^{2}$).

### Supplementary Information

**Additional file 1:** **Supplemental Table 1.** Information for each sample including: SampleID, AnimalID, Flock, Year of Birth (YOB), Sample Date, Diet, Age, Time Off Feed, Feeding, Group, Cohort, Batch, Shelf, Library and Well. **Supplemental Table 2.** Summary of Supplemental Table 1 based on Flock, Sampling Year and Cohort. **Supplemental Table 3.** Summary of Supplemental Table 1 based on Animal and Group.

**Additional file 2.** **Supplemental Table 4.** Sequencing statistics for all samples split by Group.

**Additional file 3:** **Supplemental Table 5:** Summary of the biological and systematic effects, including the variance explained by each effect, with cells coloured by effect size. **Supplemental Table 6:** Detailed parameters and results of F-tests for biological and systematic effects, along with animal and residual variance estimates.

**Additional file 4:** **Supplemental Table 7:** Heritability and repeatability estimates and standard errors for the reference-based approach. **Supplemental Table 8:** Heritability and repeatability estimates and standard errors for genera from the reference-free taxonomic assignments.

**Additional file 5.** Australian Freeze Drying. Detailed description of the Australian freeze-drying process including network diagrams visualizing the impact of freeze-drying approach on log10 normalized and Cohort-adjusted metagenome profiles.

**Additional file 6.** **Supplemental Table 9.** The genomic relationship matrix for the New Zealand samples.

## Data Availability

Raw sequence files generated and analyzed in this study are available from the NCBI Short Read Archive (SRA) database under BioProject ID PRJNA859547 (https://www.ncbi.nlm.nih.gov/bioproject/PRJNA859547). Metadata for these rumen samples are available in Additional File 1: Supplemental Table 1 and the Genomic Relationship Matrix is provided in Additional File 6.
