# Supplementary material for: Large-scale analysis of sheep rumen metagenome profiles captured by reduced representation sequencing reveals individual profiles are influenced by the environment and genetics of the host
Source: BMC Genomics. 2023 Sep 18;24:551. doi: 10.1186/s12864-023-09660-3 (PMC10506323; doi:10.1186/s12864-023-09660-3)

**Additional File 5: Freeze Drying of Australian Samples**

**Freeze Drying Approaches**

Due to the freeze drying (FD) procedure being established within the group, the 508 Australian samples were freeze dried in 5 different groups, based on different approaches to freeze drying:

**FD Approach 1 = 36 samples, FD Approach 2 = 43 samples**

Samples were defrosted in the 30 mL tubes that they were collected in and transferred to 70 mL tubes and refrozen. At the time of freeze drying, samples were taken from a -20 ^o^C freezer, lids were removed, and samples were placed in -80 ^o^C freezer for 20min prior to loading into the CHRIST Alpha 1-4 LD plus freeze drier. After the samples were in the freeze drier for a week the samples were taken out and the lids were replaced.

FD Approach 1 and FD Approach 2 used the same protocol. However, they were separated to account for potential differences in machine operation.

**FD Approach 3 = 46 samples**

Samples were taken from a -20 ^o^C freezer, lids were removed, and samples were placed in -80 ^o^C freezer for 20min prior to loading into the CHRIST Alpha 1-4 LD plus freeze drier. After the samples were in the freeze drier for a week the samples were taken out and the lids were replaced.

**FD Approach 3.5 = 15 samples**

Samples were initially loaded into the freeze drier as the top shelf, with group three but on noticing that there was some defrosting around the edge of the tubes with the samples popping up, the top shelf was removed and returned to the -20^o^C freezer. Freeze dry group 3.5 was then loaded with the group 4 samples for freeze drying.

**FD Approach 4 = 368 samples**

Lids with a large hole drilled into them were placed on the samples prior to freeze drying. Samples were loaded into the large Dynavac freeze drier (Dynapumps, Australia) at University of New England Zoology Department, directly from the freezer where the trays of tubes had been sitting on ice. Samples were dried for 14 days and airtight lids were fitted as the samples were removed from the freeze dryer.

**Confounding between Sampling Cohort and FD Approach**

Freeze drying approach was confounded with sampling cohort (e.g., all animals sampled in a given trial), with samples from sampling cohort 1 freeze dried using FD approaches 1 and 2; samples from sampling cohort 2 using FD approaches 3, 3.5 and 4; and samples from cohorts 3-7 all using FD approach 4.

**Analysis**

We wanted to investigate whether there was a strong impact of the FD approach on the rumen metagenome profile. To do this, we carried out a Network Analysis, as described in the Materials and Methods section of the main paper, with a k value of 25, reflecting the reduced number of samples for this analysis. We performed the Network Analysis on both the reference-based and reference-free metagenome profiles, with the reference-free tags considered if they were present in at least 25% of Australian samples. Network analyses were performed on the log_10_ normalized metagenome profiles as well as the cohort adjusted metagenome profiles. In this case, “cohort” represented the combination of sampling cohort and FD approach.

**Results**

Reference-based (Figure 1) and reference-free (Figure 2) Network Analyses showed similar results. There was clustering by sampling cohort in the unadjusted matrices. Any clustering by FD approach is likely to be due to sampling cohort, however, due to the confounding between sampling cohort and FD approach, there could also be differences due to FD approach. Cohort adjustment removed any evidence of samples clustering by sampling cohort or FD approach.

**Conclusions**

There is not a strong influence of FD approach on metagenome profiles and cohort adjustment removed any minor effects, therefore we can include all Australian samples in our analyses. In a typical large dataset there will be differences in the approach taken for sample handling and freeze drying, so it is promising that these effects are able to be removed by our standard adjustment.

**Figure S1: Reference Based Network Analyses**

Cohort

Freeze Drying Approach


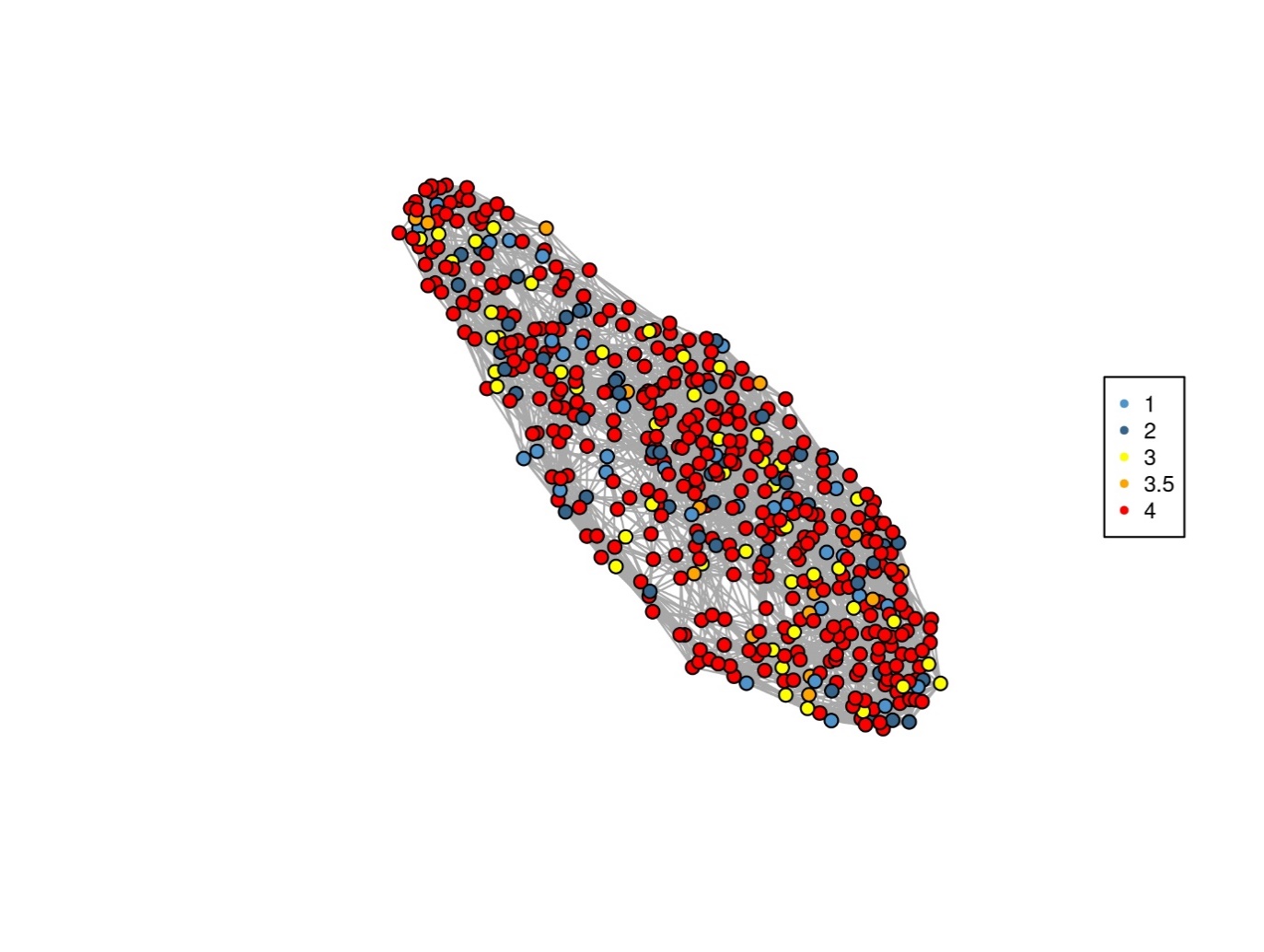

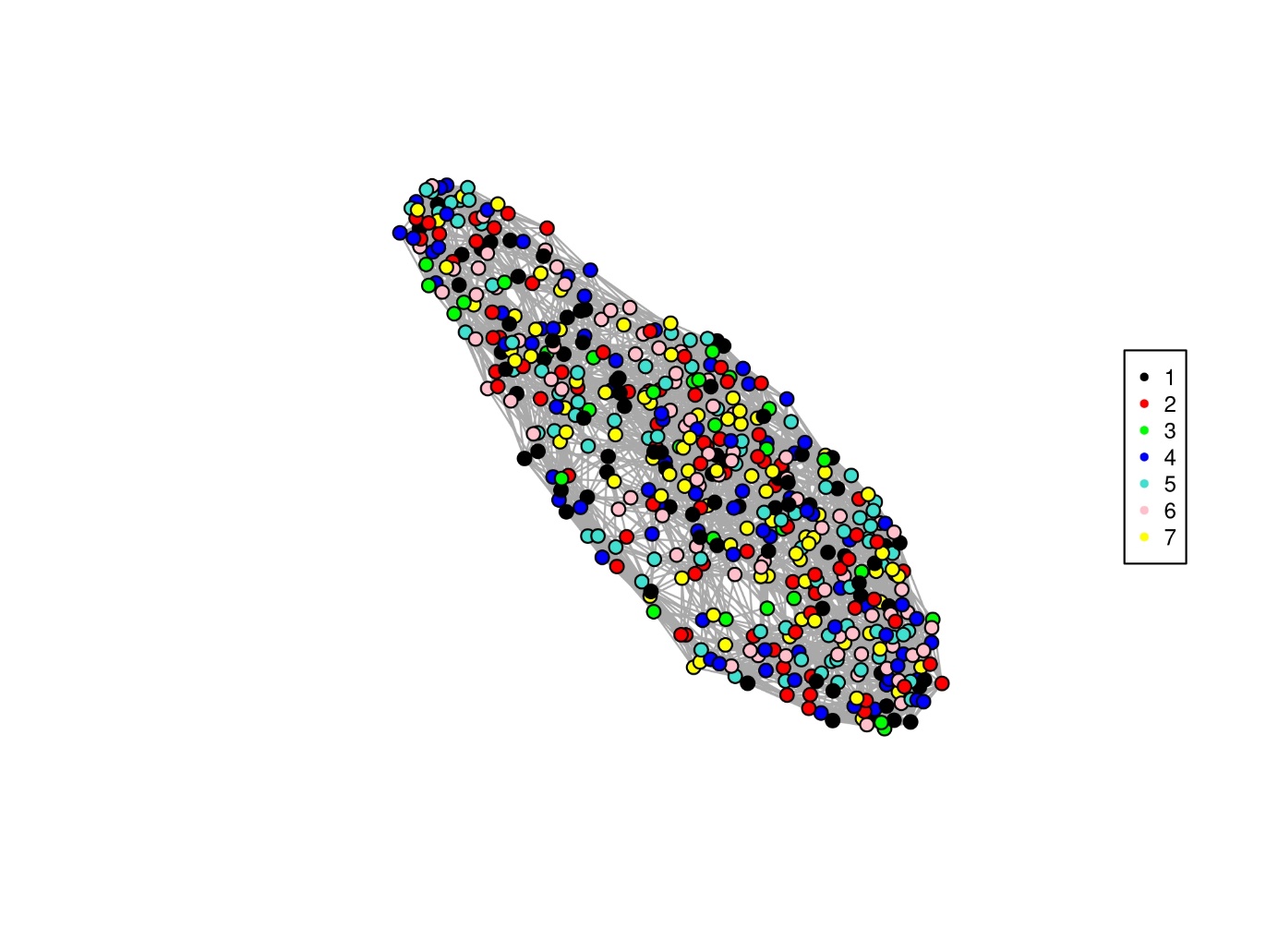

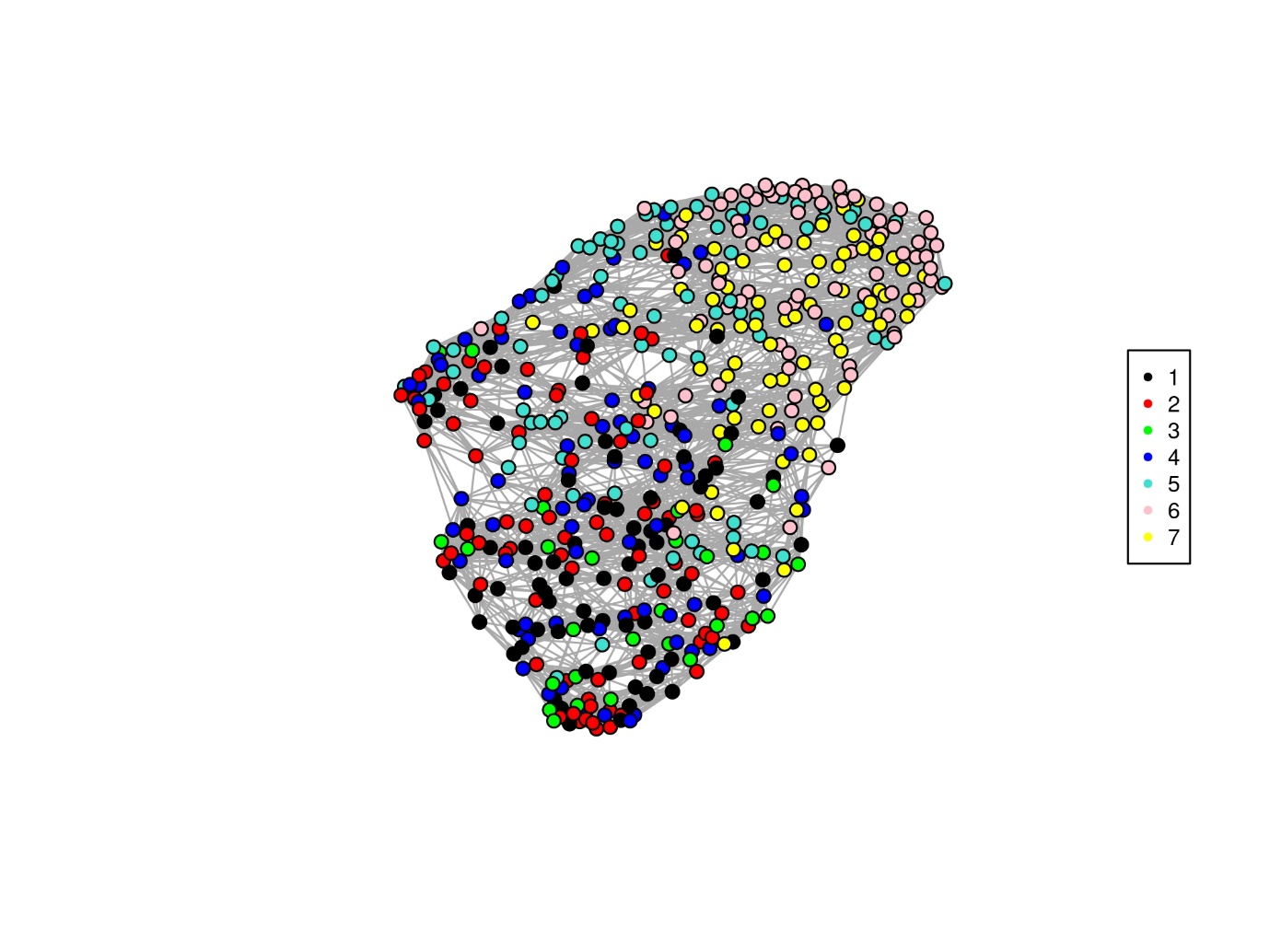

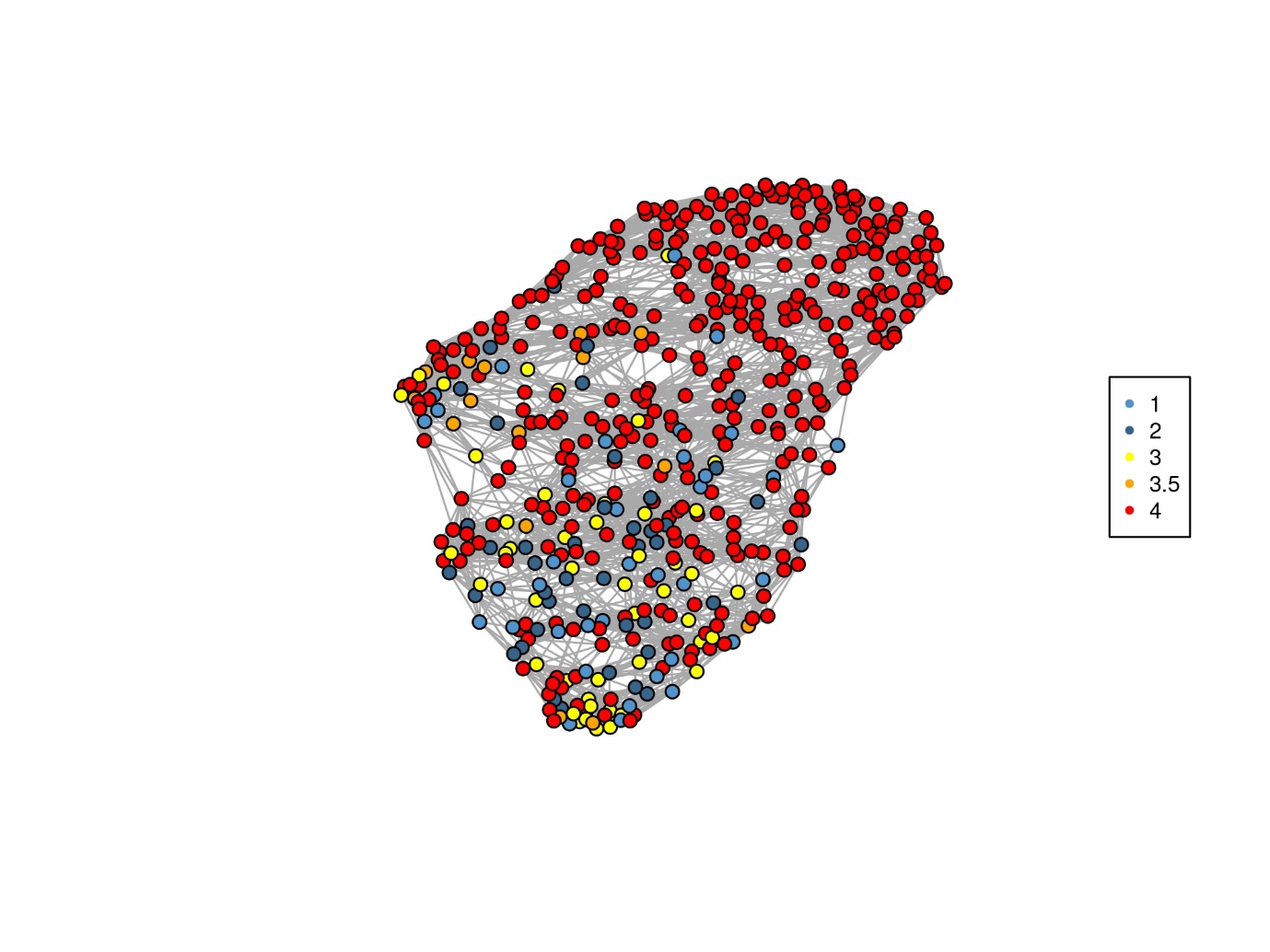


Log10 Normalized


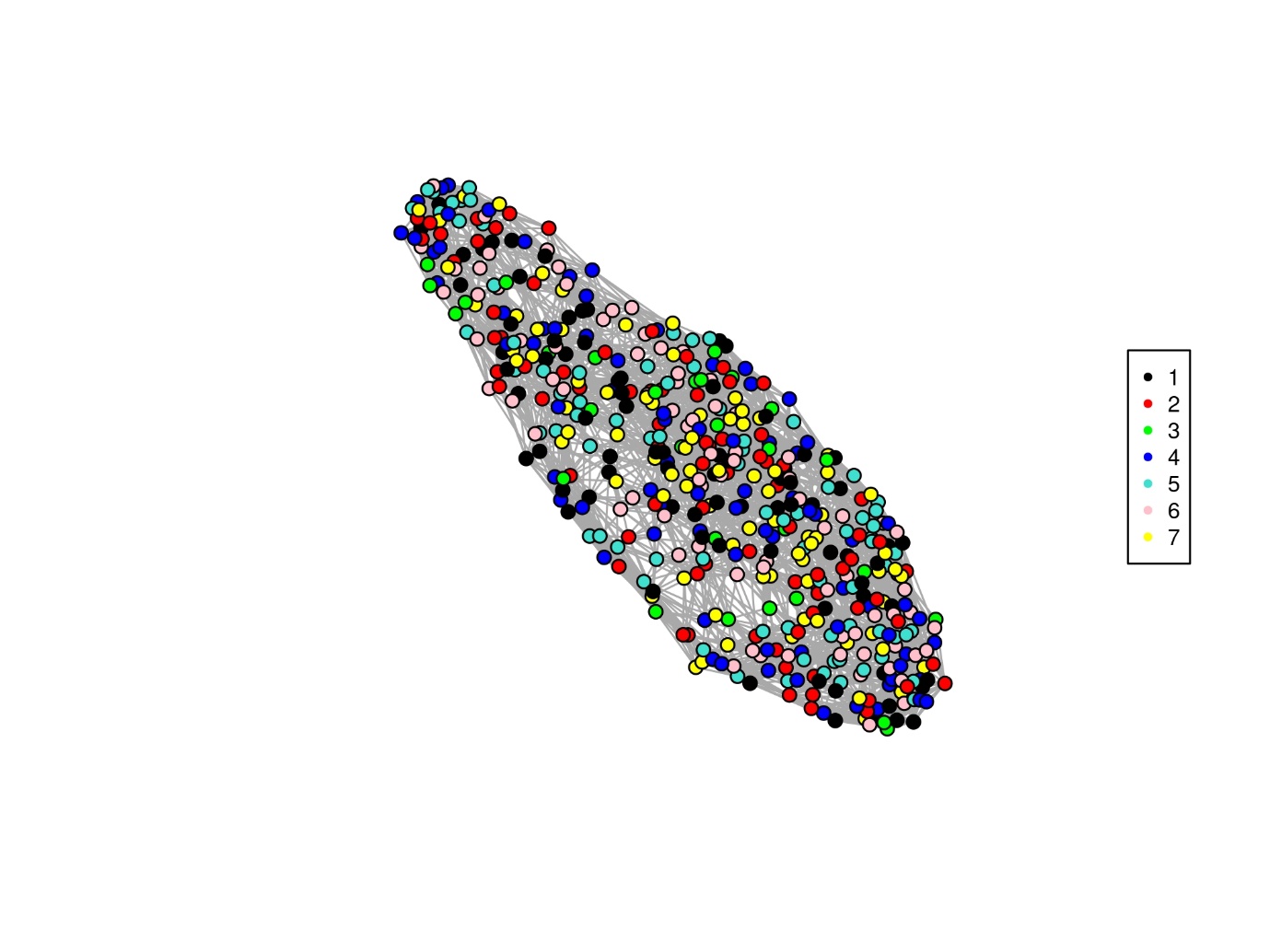

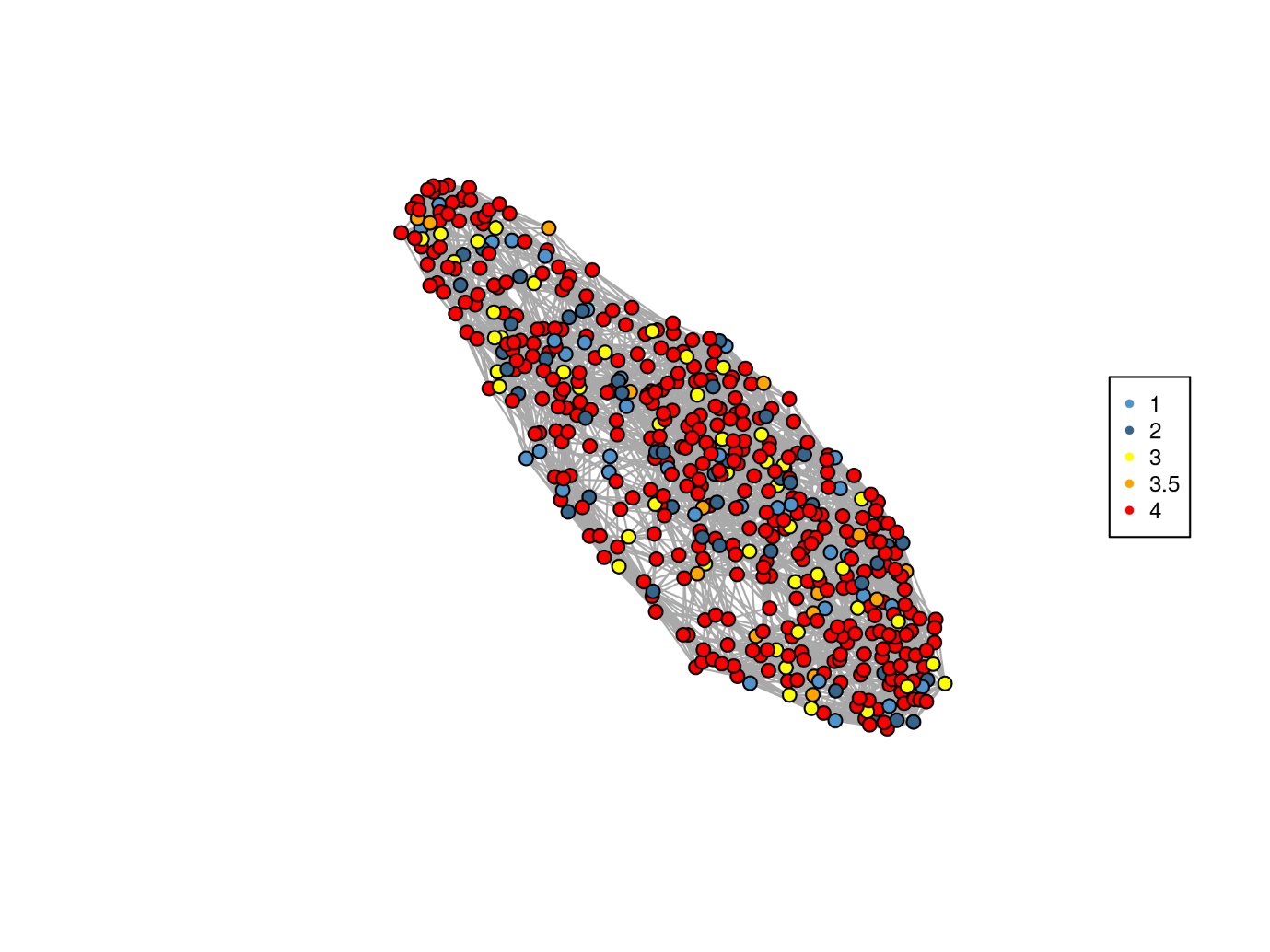


Cohort Adjusted

**Figure S2: Reference Free Network Analyses**

Cohort

Freeze Drying Approach


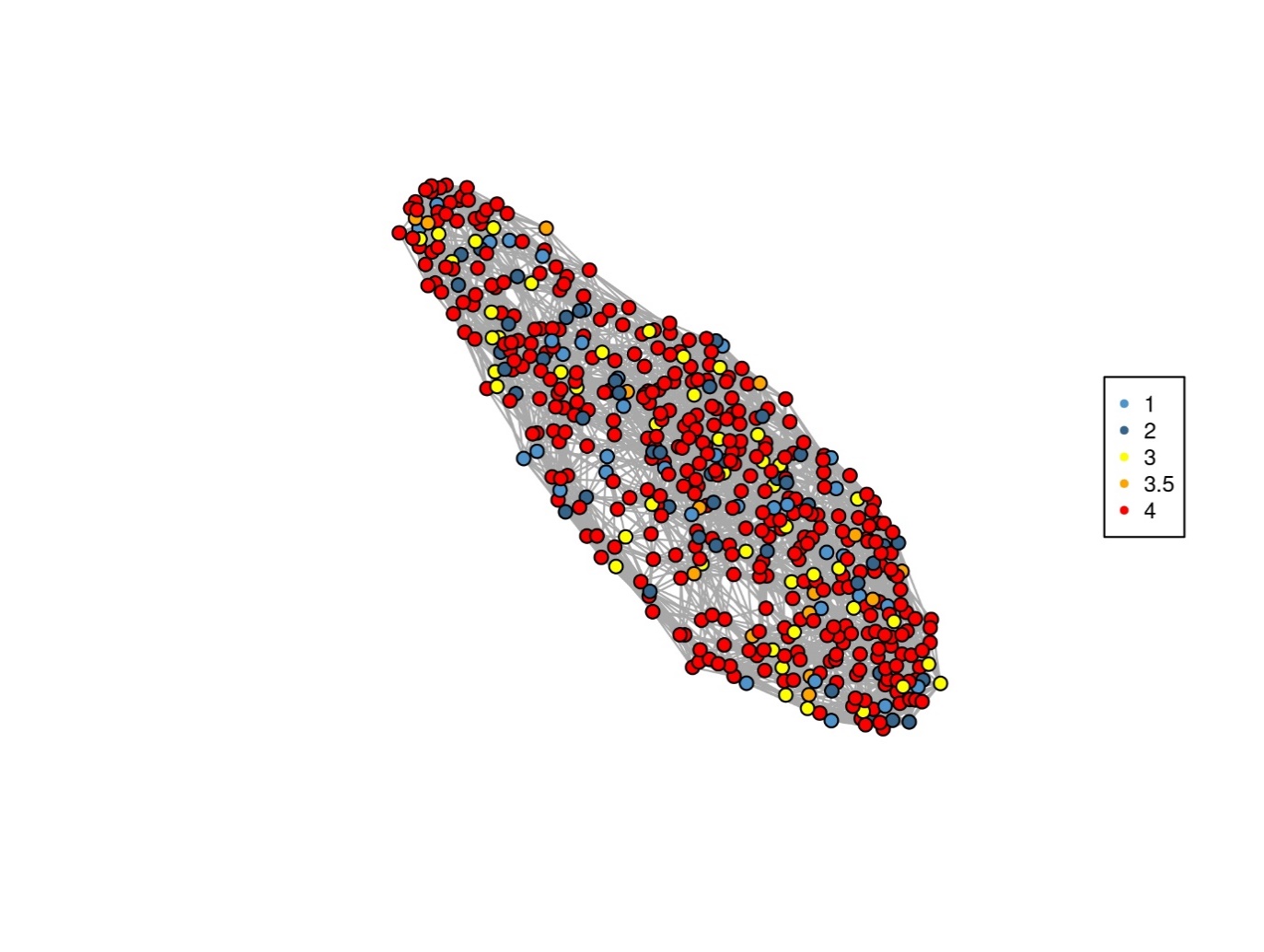

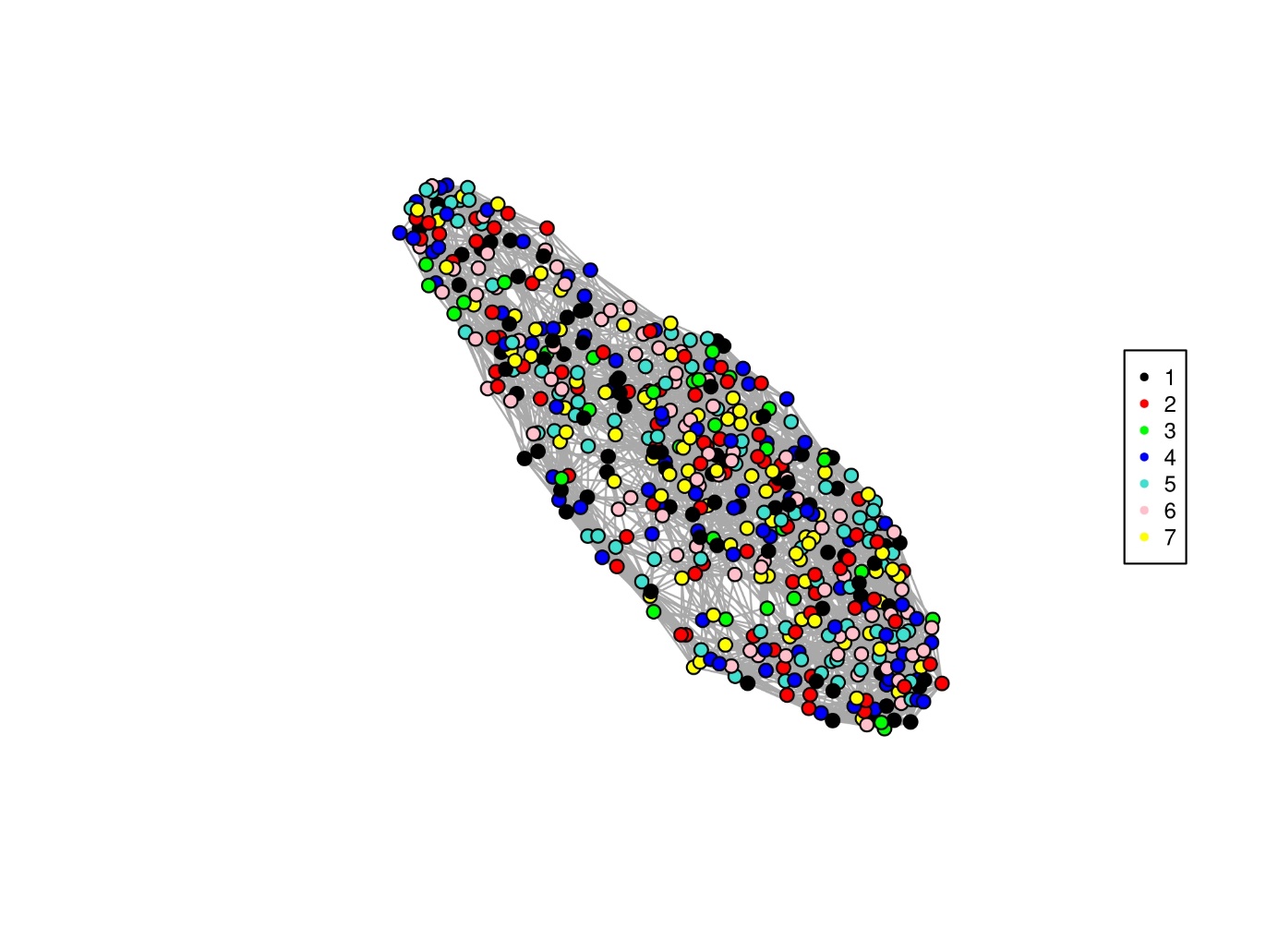

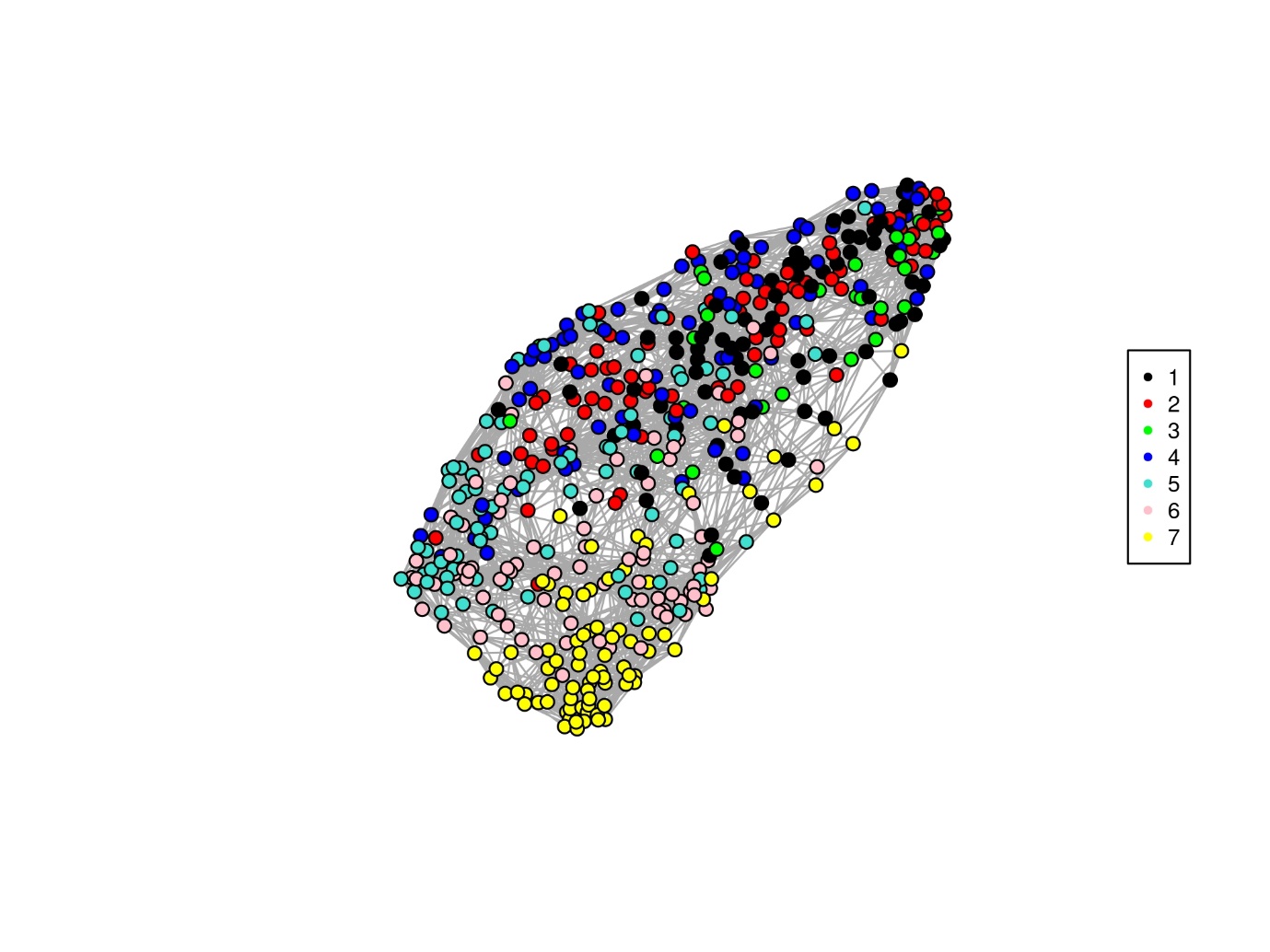

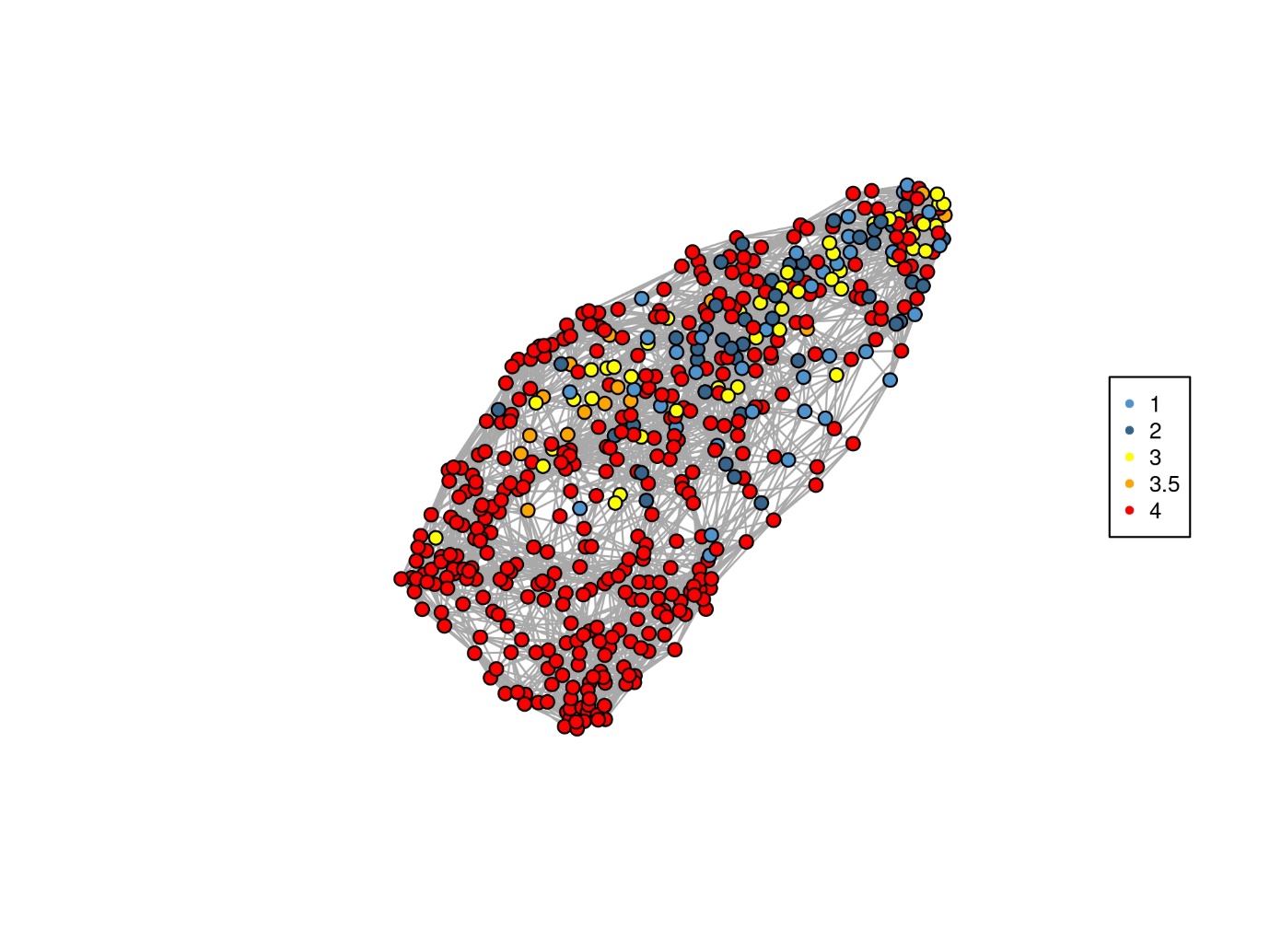


Log10 Normalized

Cohort Adjusted


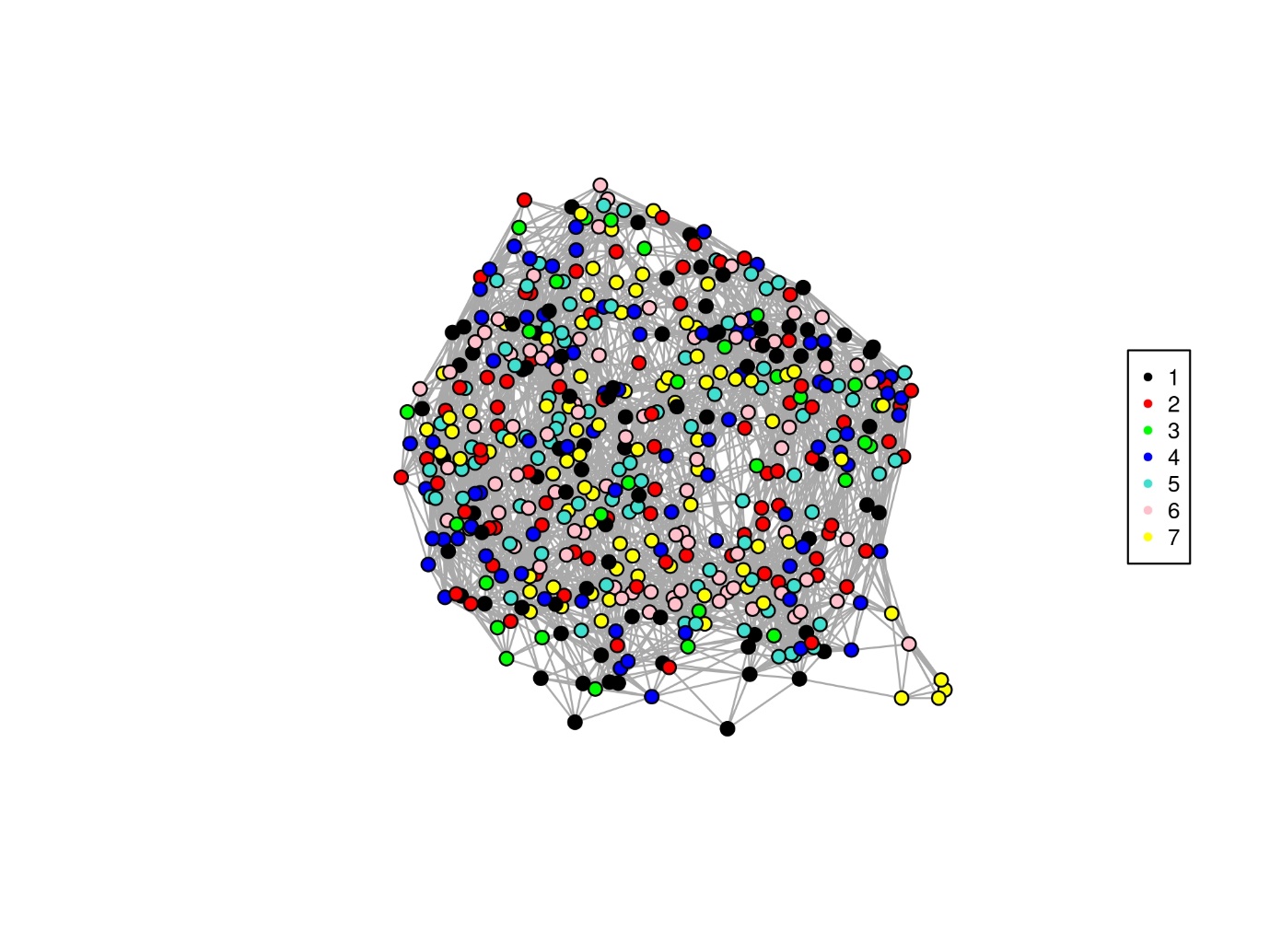

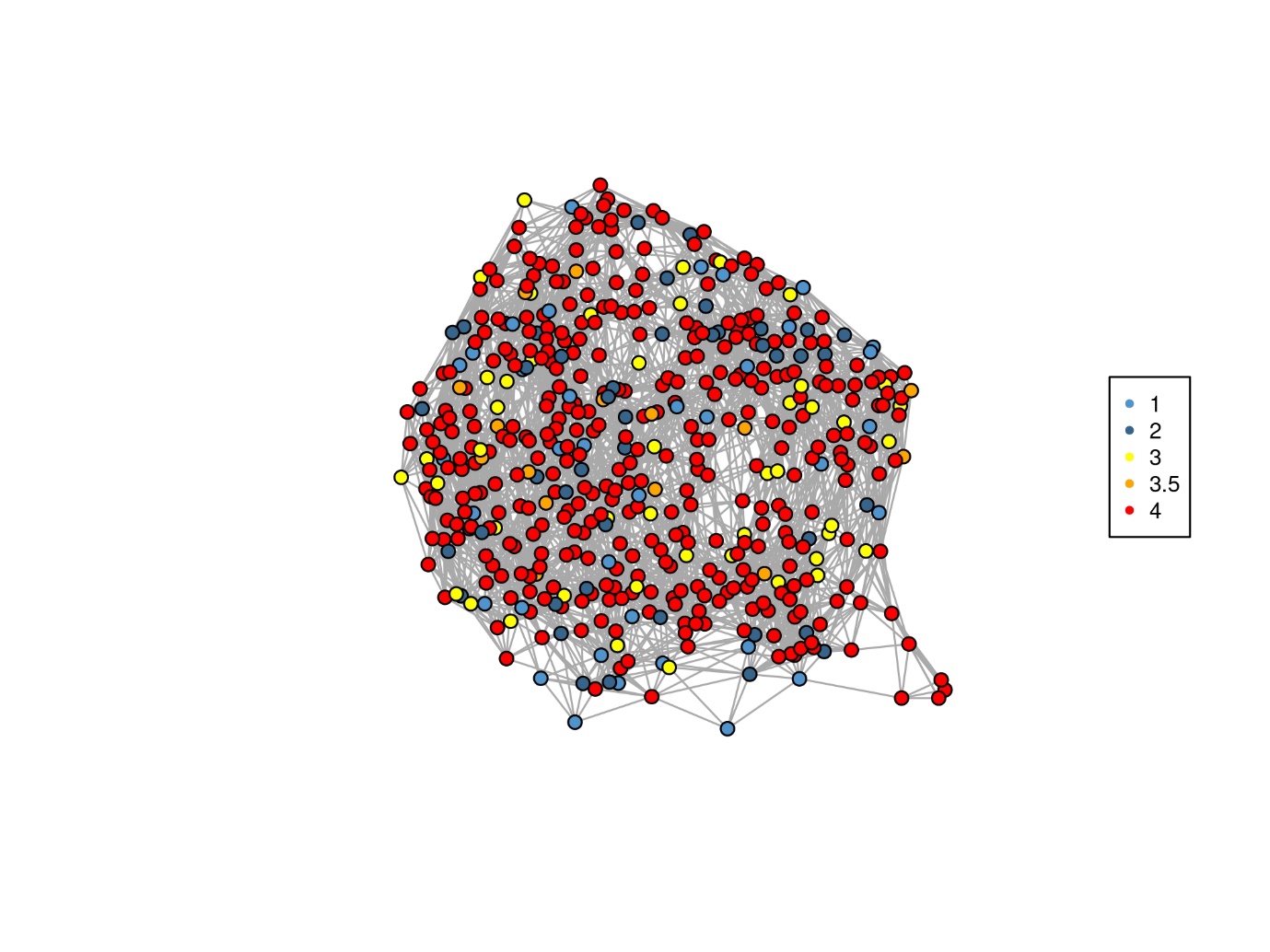

Supplement: Supplementary file 5 — Additional file 5. Australian Freeze Drying. Detailed description of the Australian freeze-drying process including network diagrams visualizing the impact of freeze-drying approach on log10 normalized and Cohort-adjusted metagenome profiles. [file 12864_2023_9660_MOESM5_ESM.docx]
